# Analyzing urban influence on extreme winter precipitation through observations and numerical simulation of two South China case studies

**DOI:** 10.1038/s41598-024-52193-2

**Published:** 2024-01-24

**Authors:** Chenxi Hu, Chi-Yung Tam, Zong-liang Yang, Ziqian Wang

**Affiliations:** 1 Earth System Science Programme, The Chinese University of Hong Kong, Hong Kong, China; 2 Shenzhen Research Institute, The Chinese University of Hong Kong, Shenzhen, China; 3 Department of Earth and Planetary Sciences, Jackson School of Geoscience, The University of Texas at Austin, Austin, TX USA; 4 School of Atmospheric Sciences, Sun Yat-Sen University, and Southern Marine Science and Engineering Guangdong Laboratory (Zhuhai), Zhuhai, China; 5 Guangdong Province Key Laboratory for Climate Change and Natural Disaster Studies, Sun Yat-Sen University, Zhuhai, China

**Keywords:** Atmospheric dynamics, Natural hazards

## Abstract

This study investigates the impact of urbanization on extreme winter rainfall in the South China Greater Bay Area (GBA) through the analysis of hourly station observations and simulations using the Weather Research and Forecasting Model with the Single Layer Urban Canopy Model (WRF-SLUCM). Data from 2008 to 2017 reveal that urban areas in the GBA experience lower 99th percentile hourly winter rainfall intensity compared to surrounding rural regions. However, urban locations exhibit higher annual maximum hourly rainfall (Rmax) and very extreme rainfall events (99.99th percentile) in winter, suggesting a positive influence of urbanization on extreme winter precipitation. A case study further underscores the role of the Urban Heat Island (UHI) effect in enhancing extreme rainfall intensity and probability in the GBA urban areas. Additionally, two extreme cases were dynamically downscaled using WRF-SLUCM, involving four parallel experiments: replacing urban land use with cropland (Nourban), using historical urban land use data from 1999 (99LS), projecting near-future urban land use for 2030 (30LS), and considering 2030 urban land use without anthropogenic heat (AH) (30LS-AH0). Synoptic analysis demonstrates that cold air intrusion suppresses the GBA UHI in Case 2013 but not in Case 2015. Reduced evaporation and humidity induced by urban surfaces significantly decrease urban precipitation in Case 2013. In contrast, the persistent UHI in Case 2015 enhances local convection and land–ocean circulation, leading to increased moisture flux convergence and amplified urban precipitation intensity and probability in 30LS compared to Nourban. This amplification is primarily attributed to AH, while the change in 99LS remains insignificant. These findings suggest that urban influences on extreme precipitation in the GBA persist during winter, particularly when the UHI effect is maintained.

## Introduction

Over the past century, rapid urbanization has significantly altered the global demographic landscape, leading to an approximate 80% expansion of urban areas between 1985 and 2015, equivalent to a yearly increase rate of 9687 km^2^^1^. This swift urban expansion has brought about substantial changes in local temperature, precipitation, and atmospheric circulation within cities^2–6^. Urban areas are known to absorb and store more shortwave radiation due to factors such as modified land use, reduced surface albedo, and distinctive urban morphology, thereby enhancing surface temperatures^7–11^. Additionally, the release of anthropogenic heat (AH) from buildings, traffic, and human activities fosters the formation of urban heat islands (UHIs)^12,13^, UHIs result in higher surface temperature^2^ and an environment conducive to convection^14,15^. The prevalence of impervious urban land surfaces leads to reduced evaporation, water vapor content, and convective available potential energy (CAPE)^16–21^. However, the impact of urbanization on global precipitation patterns is complex and varies depending on geographical location and UHI intensity^22–28^, with a consensus yet to be reached.

The Greater Bay Area (GBA) mega-city cluster, located along the South China (SC) coast (covering 22–23.2° N, 112.6–114.3° E), has experienced rapid urbanization since the early 1980s and has grown into one of the world's largest mega-city clusters. Previous research has emphasized the substantial intensification of extreme precipitation in the GBA due to urbanization, primarily driven by the UHI effect^28–34^. However, most of these studies focus on annual or summertime monsoon precipitation.

During the winter season, numerous studies have investigated the UHI intensity in the GBA mega-city. Winter warming induced by UHI effects in the Guangzhou metropolitan area has been observed to surpass that resulting from global warming, primarily due to industrial and transportation activities^35^. Observations indicate that the urban–rural air temperature difference in winter can be comparable to that in summer, reaching 0.4–1.8 °C in major GBA mega-cities from 2000 to 2017^36^. Remote sensing data also indicate an even stronger UHI intensity in winter than in summer for the Guangzhou metropolitan area^37^. Numerical simulations further reveal that urbanization amplifies upward surface sensible heat flux, increasing air temperatures in GBA mega-cities by approximately 0.5–2 °C, particularly in the highly urbanized northwestern regions^3^. Overall, the GBA mega-city experiences a significant UHI effect during winter, which can potentially influence the local climate, particularly in terms of extreme precipitation.

In addition, extreme winter precipitation in coastal SC, influenced by the East Asian winter monsoon, holds substantial consequences for local agriculture, transportation, human lives, and the economy^38^. Winter precipitation accounts for over 10% of annual rainfall in SC, with heavy rainfall persisting despite less favorable weather conditions compared to summer^39,40^. Multiple studies have explored the relationship between SC winter rainfall and various factors, including the synoptic systems, large-scale circulation over the western North Pacific, sea surface temperature (SST) in the SCS, El Niño-Southern Oscillation (ENSO), and winter monsoon^41–46^. Nevertheless, the potential impacts of rapid urban development in the GBA on local extreme winter rainfall remain inadequately understood.

Generally, the atmosphere is drier in winter compared to summer, leading to stronger urban drying island (UDI) effects in coastal cities during the winter months^47,48^. This phenomenon can decrease local precipitation by reducing local moisture content in urban areas^20–23,49,50^. For example, winter precipitation in downtown and southern Beijing decreased by approximately 19% during 1981–2000 compared to the 1961–1980 period^51^. Similar results have been observed in GBA, where urban land surfaces tend to reduce accumulated winter precipitation in GBA cities due to drier northerly winds, decreased latent heat, and enhanced atmospheric stability^52,53^.

Despite these findings, most studies have primarily focused on long-term climatic accumulated winter precipitation, leaving the impact of urbanization on extreme winter rainfall characteristics under different weather conditions largely unexplored. Given the significant UHI in the GBA mega-city cluster during winter and the importance of winter precipitation, it is crucial to investigate the potential impacts of urbanization on extreme winter precipitation in this region. To address this knowledge gap, this study aims to answer two key questions: (1) how urbanization, including the UHI effect and urban land use, affects winter rainfall characteristics in GBA mega-cities, and (2) whether urban effects on winter heavy rainfall differ under different synoptic conditions. These objectives will be achieved through (1) an examination of the climatology of winter precipitation in GBA and the impact of UHI on extreme winter rainfall based on station observations, and (2) the utilization of WRF-SLUCM to dynamically downscale two specific winter extreme events characterized by distinct weather conditions. Through various model experiments, this research seeks to unravel the impacts of urban surface land use and UHI on extreme winter rainfall in GBA mega-cities. These findings can help bridge gaps in our understanding of extreme winter precipitation and urban climate in the GBA, providing valuable insights for policymakers involved in agriculture, transportation planning, public policy formulation. Ultimately, this knowledge can enhance the resilience of urban communities in the face of future extreme events.

## Results

### Observation results

#### Winter temperature and precipitation

**Figure 1:**
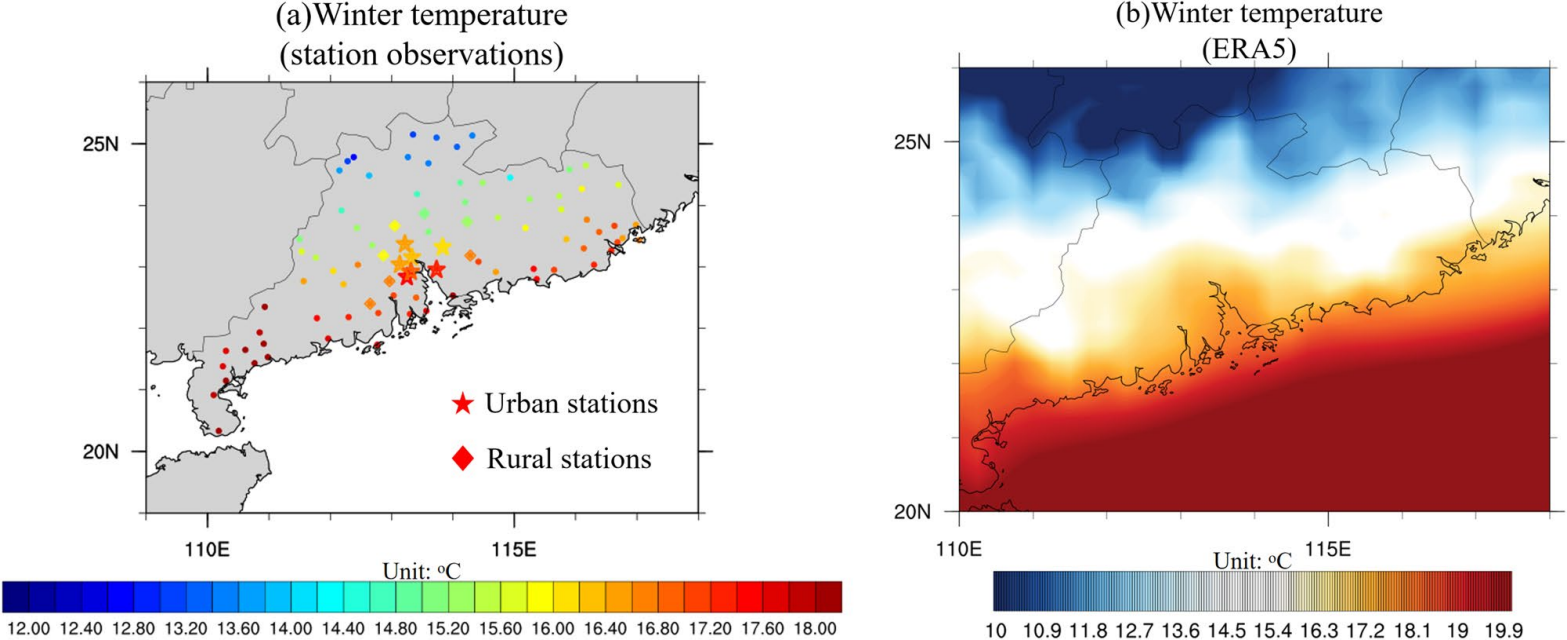
(**a**) Near-surface temperature (units: °C) averaged over winter from 2008 to 2017, with star and diamond meaning urban and rural stations. (**b**) Winter near-surface temperature (units: °C) derived from ERA5 reanalysis data. Black contours mean the coastline and boundary of province. The maps were generated by the NCAR Command Language (NCL) Version 6.6.2^79^, http://dx.doi.org/10.5065/D6WD3XH5.

Figure 1 shows the winter near-surface temperatures averaged from 2008 to 2017, including results from (a) observations and (b) ERA5 reanalysis data. Winter near-surface temperatures exhibit a discernible decline from south to north, with maximum temperatures surpassing 18 °C in the southern part of SC (Leizhou Peninsula) and only reaching 12–13 °C at inland stations located at 25° N, the magnitude of this temperature gradient is considerably stronger than that observed in spring and summer. Coastal areas also exhibit warmer temperatures than their inland counterparts. When comparing urban and surrounding rural stations, winter near-surface temperatures at urban stations range from 16.5 to 17.5 °C, while rural stations exhibit temperatures only between 15 and 16.8 °C. From 2008 to 2017, the average temperature difference between all urban and rural stations was 1.02 °C. Results from ERA5 are consistent with observations, indicating higher temperatures in the SCS area compared to land areas during winter, with ocean near-surface temperatures exceeding 20 °C. Near-surface temperatures in the SC land area gradually decrease from 19 °C in the coastline to below 10 °C at 25° N. Moreover, the GBA exhibits higher temperatures than surrounding rural areas, with temperatures in the GBA mega-city ranging from 17 to 18.5 °C, while temperatures in adjacent rural areas vary between 15 and 17.5 °C. The consistent observation of higher temperatures in urban areas compared to surrounding rural regions indicates a strong UHI intensity during winter in the GBA^3,36,37^. This finding highlights the significance of UHI on local climate in winter under rapid urbanization, and underscores the need for further research to understand the implications of urbanization on extreme winter precipitation in GBA mega-city.

**Figure 2:**
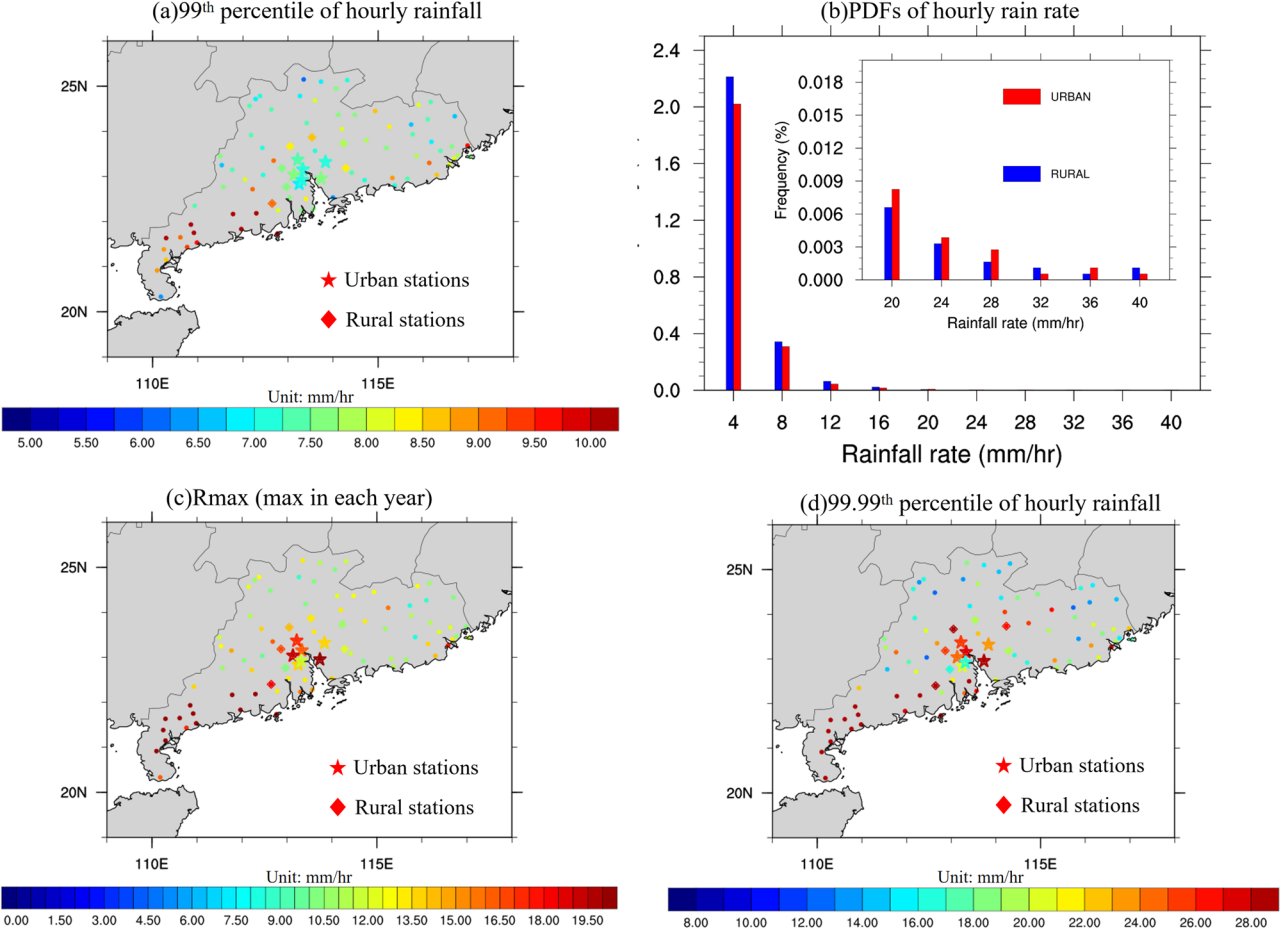
(**a**) Extreme rainfall based the 99th percentile of hourly rainfall (units: mm/h) during winter for each station, with star meaning urban stations, diamond meaning rural stations, and dots other stations. (**b**) PDFs of hourly precipitation rates during winter for rural (blue) and urban (red) stations, within the ranges of 1–4, 4–8, 8–12, 12–16, 16–20, 20–24, 24–28, 28–32, 32–36, and 36–40 mm/h. (**c**,**d**) Same as (**a**) except for (**c**) Rmax (units: mm/h), annual maximum value in winter averaged from 2008 to 2017 in each station, and (**d**) Extreme rainfall based 99.99th percentile of hourly rainfall (units: mm/h) during winter. The maps were generated by the NCAR Command Language (NCL) Version 6.6.2^79^, http://dx.doi.org/10.5065/D6WD3XH5.

Analysis of precipitation in SC uncovers distinct patterns in extreme rainfall intensity during winter. Figure 2a shows the hourly extreme winter rainfall intensity, based on the 99th percentile of hourly rainfall in winter over the 10-year period (2008–2017) in SC derived from station observations. Seven urban and seven rural stations are represented by stars and diamonds, respectively, while the other stations are represented by dots. Extreme rainfall intensities during winter are highly variable, with only the southern part of SC experiencing intensities above 10 mm/h. Interestingly, urban areas exhibit lower extreme rainfall intensities, ranging from 6.5 to 7.75 mm/h, compared to surrounding rural areas with intensities of 8.5 mm/h or more. This is completely different from the extreme rainfall distribution in summer and spring in SC^33,34,54,55^. Also shown is the Probability Density Functions (PDFs) of hourly rainfall for urban and rural stations (Fig. 2b). Rainfall probability in winter is generally lower than in other seasons, with frequencies of 1–10 mm/h rainfall occurring less than 3% for both urban and rural locations. Notably, light rain (< 16 mm/h) is more probable in rural areas, with a decrease of about 20% to 5% in the 1–16 mm/h range compared to urban areas. However, heavy rainfall (> 16 mm/h) is more likely in urban locations, with a 10% to 30% increase in the 16–28 mm/h range compared to rural areas. These observations suggest that the lower evaporation and humidity of urbanization may contribute to lower intensity and probability of rainfall in the GBA mega-city compared to surrounding rural areas. Nevertheless, more frequent intense rainfall events exceeding 16 mm/h still occur in urban regions, implying potential positive urban impact on very heavy rainfall in winter.

To further investigate the urban impact on very extreme winter precipitation, also shown in Fig. 2 are the (c) mean value of Rmax (annual maximum precipitation) in winter from 2008 to 2017, and (d) the 99.99th percentile of winter hourly rainfall in SC derived from station observations. The distribution of Rmax and 99.99th percentile of hourly rainfall aligns with extreme rainfall patterns (see Fig. 2a), with the southern part of SC receiving the highest rainfall. Notably, urban stations, particularly Guangzhou (113.29° E, 23.13° N), Foshan (113.15° E, 22.51° N), and Dongguan (113.44° E, 22.58° N), exhibit substantially stronger Rmax intensities than their rural counterparts, reaching over 20 mm/h, while those in rural stations range only about 7–16 mm/h. Similarly, the 99.99th percentile of hourly precipitation is more intense in GBA urban areas, ranging from 18 to over 30 mm/h, compared to 12–25 mm/h in rural locations. Consequently, while the 99th percentile of hourly rainfall is suppressed in urban areas during winter, very extreme rainfall events like Rmax and the 99.99th percentile of hourly rainfall remain notably stronger in urban areas compared to rural areas, indicating positive urban impact on extreme winter rainfall in GBA mega-city.

#### Case study for UHI effect on winter precipitation

A total of 35 cases were selected based on the 99th percentile of extreme winter rainfall for urban and rural stations, with 11 (12) Strong (weak) UHI cases further classified (see section "Observations and winter case study"). Both strong and weak UHI cases exhibit a gradual decrease in GBA near-surface temperatures during rainfall events (see Fig. S1), which is typically associated with cold air intrusion during winter rainfall. Prior to the hour of peak rainfall averaged over the 14 selected stations, the urban–rural near-surface temperature difference is 0.8–1.1 °C for strong UHI cases, but only 0.3–0.5 °C for weak UHI cases. The onset of extreme rainfall suppresses the UHI in both cases, reducing the urban–rural temperature difference to 0.4–0.7 °C for strong UHI cases and 0.2–0.5 K for weak UHI cases after the hour of peak rainfall.

**Figure 3:**
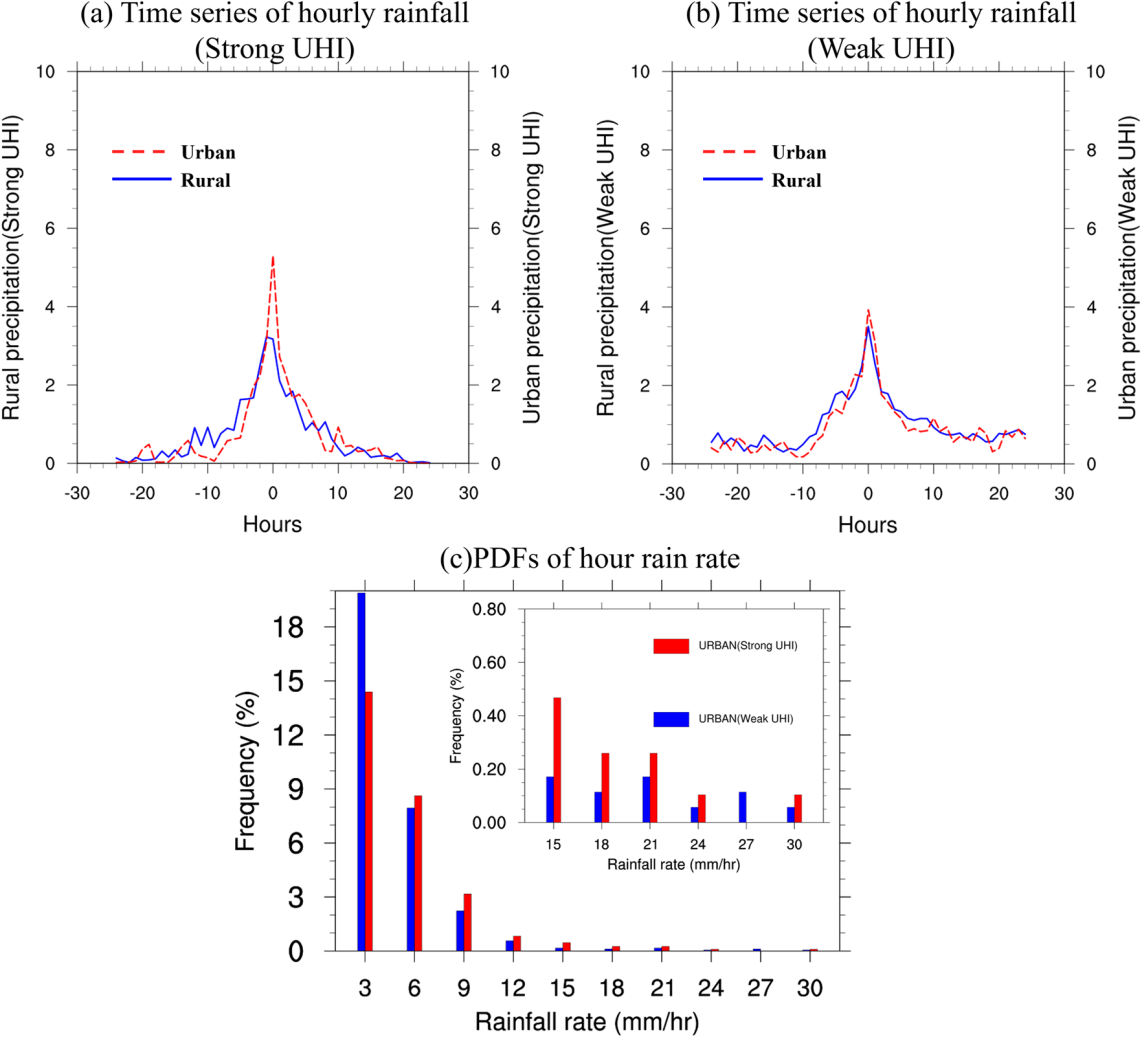
(**a**) Time series of hourly rainfall (units: mm/h) averaged over urban and rural stations during all strong UHI cases, with red and blue line representing rainfall in urban and rural stations. Time = 0 refers to the peak rainfall hour averaged over the selected 14 stations. (**b**) Same as (**a**) except for weak UHI cases. (**c**) PDFs of hourly precipitation rates in urban stations for weak UHI (blue) and strong UHI (red) cases, within the ranges of 1–3, 3–6, 6–9, 9–12, 12–15, 15–18, 18–21, 21–24, 24–27, and 27–30 mm/h.

A case study investigates the impact of UHI on extreme winter precipitation across urban and rural locations in the GBA by analyzing 11 strong and 12 weak UHI cases. The composite time series of hourly rainfall is presented in Fig. 3 for (a) strong UHI cases, and (b) weak UHI cases, with t = 0 h denoting the time of peak rainfall averaged over the 14 selected stations for entire cases. For strong UHI cases, a higher precipitation intensity is observed in rural areas from t = − 12 h to t = − 5 h compared to urban areas, which could be attributed to the placement of some rural stations to the north of the city. These rural locations experience more precipitation before the peak hour due to the prevalence of frontal-related winter precipitation from the north. However, at t = 0 h, the rainfall intensity in urban locations reaches 5.5 mm/h, while it amounts to only about 3.2 mm/h in rural areas. Urban areas exhibit stronger precipitation from t = − 2 h to t = 7 h in comparison to rural areas. For weak UHI cases, similar patterns emerge, with higher rainfall in rural areas preceding the peak hour. However, the rainfall difference between urban and rural locations remains marginal at t = 0 h, with urban and rural areas experiencing 4 mm/h and 3.6 mm/h, respectively. This difference rapidly diminishes to negative values after t = 2 h. These findings indicate that precipitation differences between urban and rural areas are more pronounced in strong UHI events than in weak ones, implying that the UHI can influence extreme winter precipitation intensities in the GBA mega-city. The PDFs of hourly rainfall over urban stations for strong and weak UHI cases are depicted in Fig. 3c. Although the probability of very light rainfall (1–3 mm/h) decreases by more than 20% in strong UHI cases compared to weak ones, the hourly urban rainfall probability across nearly all ranges (except 24–27 mm/h) is higher in strong UHI cases. For rainfall intensities between 3 and 24 mm/h, the probability increases by approximately 5% to over 200%, particularly for hourly rainfall exceeding 15 mm/h, which is considered extremely heavy rainfall during winter in GBA. Overall, the observations indicate that UHI may still enhance the intensity and likelihood of extreme rainfall events in GBA urban areas, even though urban areas generally receive less accumulated winter precipitation than rural areas due to the lower evaporation and humidity of urban land surface.

### Results based on numerical modeling

#### Synoptic background for two cases

**Figure 4:**
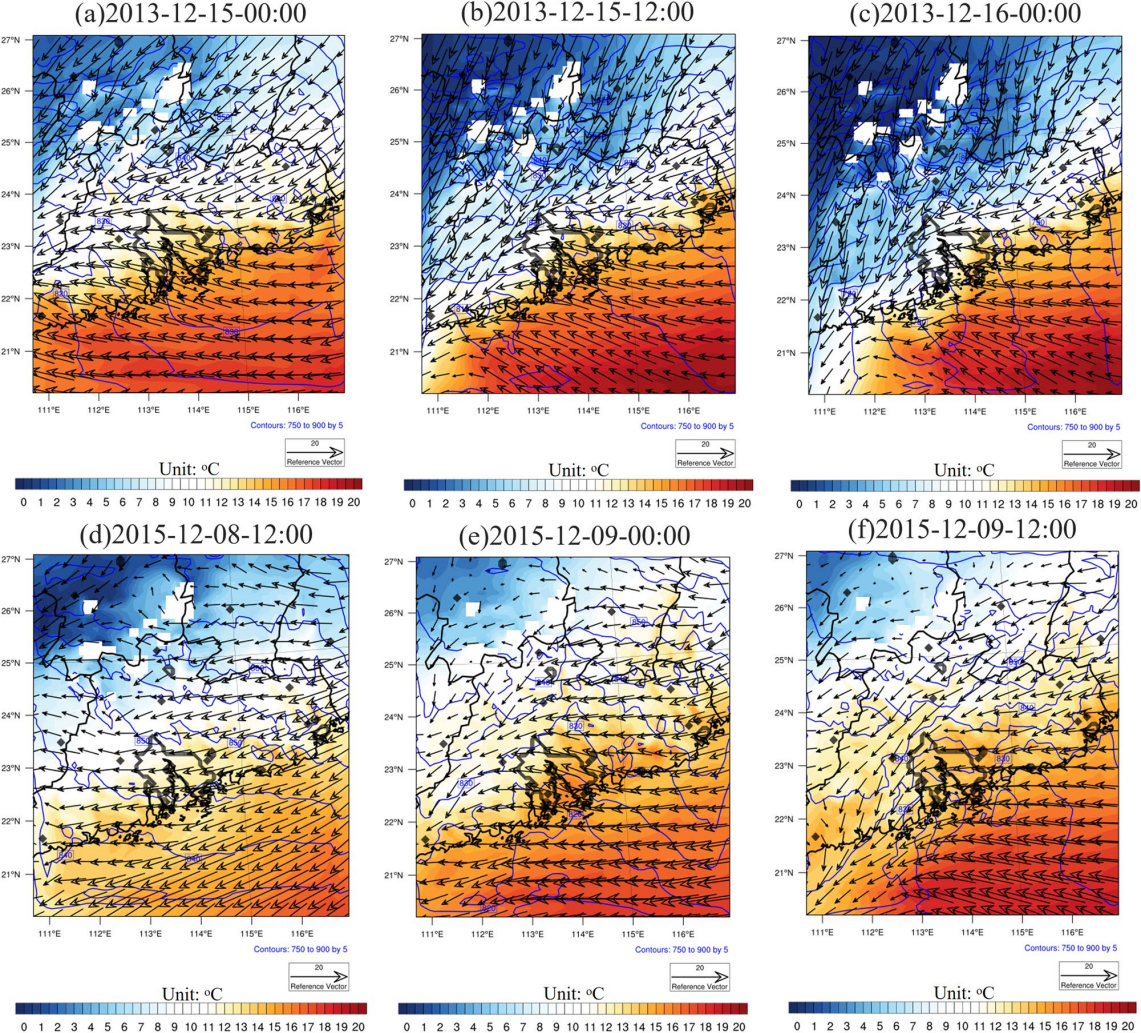
Snapshot of 925 hPa temperature (units: °C), geopotential height (units: m), and U,V wind (units: m/s) for Case 2013 from ERA5 reanalysis data at (**a**) 2013–12-15–00:00, (**b**) 2013–12-15–12:00, and (**c**) 2013–12-16–00:00, with shaded, blue contour, and vector representing temperature, geopotential height, and wind. Black contours represent coastline, boundary of province, and boundary of GBA urban areas. (**d**–**f**) Same as (**a**–**c**) except for Case 2015. The maps were generated by the NCAR Command Language (NCL) Version 6.6.2^79^, http://dx.doi.org/10.5065/D6WD3XH5.

Based on the 99.9th percentile of hourly rainfall in GBA, two distinct extreme winter rainfall events were selected and dynamically downscaled within the SC region and GBA (see section "WRF model configuration and two winter cases"). Figure 4 shows the snapshots of model-derived low-level (925 hPa) temperatures, U and V wind components, and geopotential heights during days of peak rainfall in GBA for Case 2013 and Case 2015 (refer to the time in the figure title), with black contours delineating coastline, provincial boundaries, and GBA urban areas. For Case 2013, it is evident that the prevailing low-level winds north of SC are northerly before the rainfall, while SC experiences easterly winds. A substantial temperature gradient exists from north to south, with temperatures ranging from 1 to 16 °C, and ocean 925 hPa temperatures lying between 16 and 19 °C. The geopotential height gradient over SC is relatively relaxed. However, during the rainfall event, cold air infiltrates SC, increasing the temperature gradient and causing negative temperatures in the northern part of SC. On December 16th, a stronger northerly wind emerges over most SC landmass, replacing the prevailing easterly wind. This northerly wind, associated with the southward progression of cold air towards the SC coastline, forces easterly winds to retreat to the coast and ocean, resulting in a strong cold front over SC. And the 925 hPa temperature within the GBA urban area drops substantially from 12 to 16 °C on Dec 15th to only 5–12 °C on Dec 16th. Concurrently, the geopotential height gradient becomes much denser in the SC area during rainfall events, corresponding to the rapid movement of cold air and enlarged temperature gradient. The invasion of cold air and the approach of cold fronts primarily contribute to the extreme winter precipitation in the western part of SC for Case 2013.

Similarly, Fig. 4d–f display snapshots of low-level (925 hPa) temperature, U and V winds, and geopotential height derived from model outputs during the day of peak rainfall for Case 2015. At 12:00 p.m. on December 8th, the predominant wind direction across SC region is easterly, with strong northeasterly winds over the ocean. A cold center with temperatures around 0–2 °C is situated north of SC, accompanied by weak northeasterly winds. During the extreme rainfall event, the cold air does not migrate southward to SC, although the northeasterly winds strengthen over time. 925 hPa temperatures within the GBA mega-city range from 12 °C to more than 17°C throughout the rainfall process, while the cold air center remains stationed north of SC. Nonetheless, strong northeasterly winds persist over the northern part of SC during the rainfall, flowing into potent easterly winds over the ocean and forming a convergence zone and surface front along the GBA. This front coincides with the location of extreme rainfall in the southern part of SC. It is also worth noting that the geopotential height gradient remains loose on December 9th, corresponding to a weaker and relatively stable temperature gradient in SC for Case 2015 compared to Case 2013.

In summary, surface fronts induced by northerly winds from land and easterly winds from the ocean lead to extreme rainfall in GBA cities for both Case 2013 and Case 2015. However, the synoptic backgrounds of the two events differ; in Case 2015, cold air does not dominate the cities, resulting in varying temperatures within the GBA compared to Case 2013. Consequently, further research will investigate (1) the potential role of urban impact on extreme winter rainfall in GBA, and (2) whether this influence varies under different synoptic backgrounds.

#### Temperature

**Figure 5:**
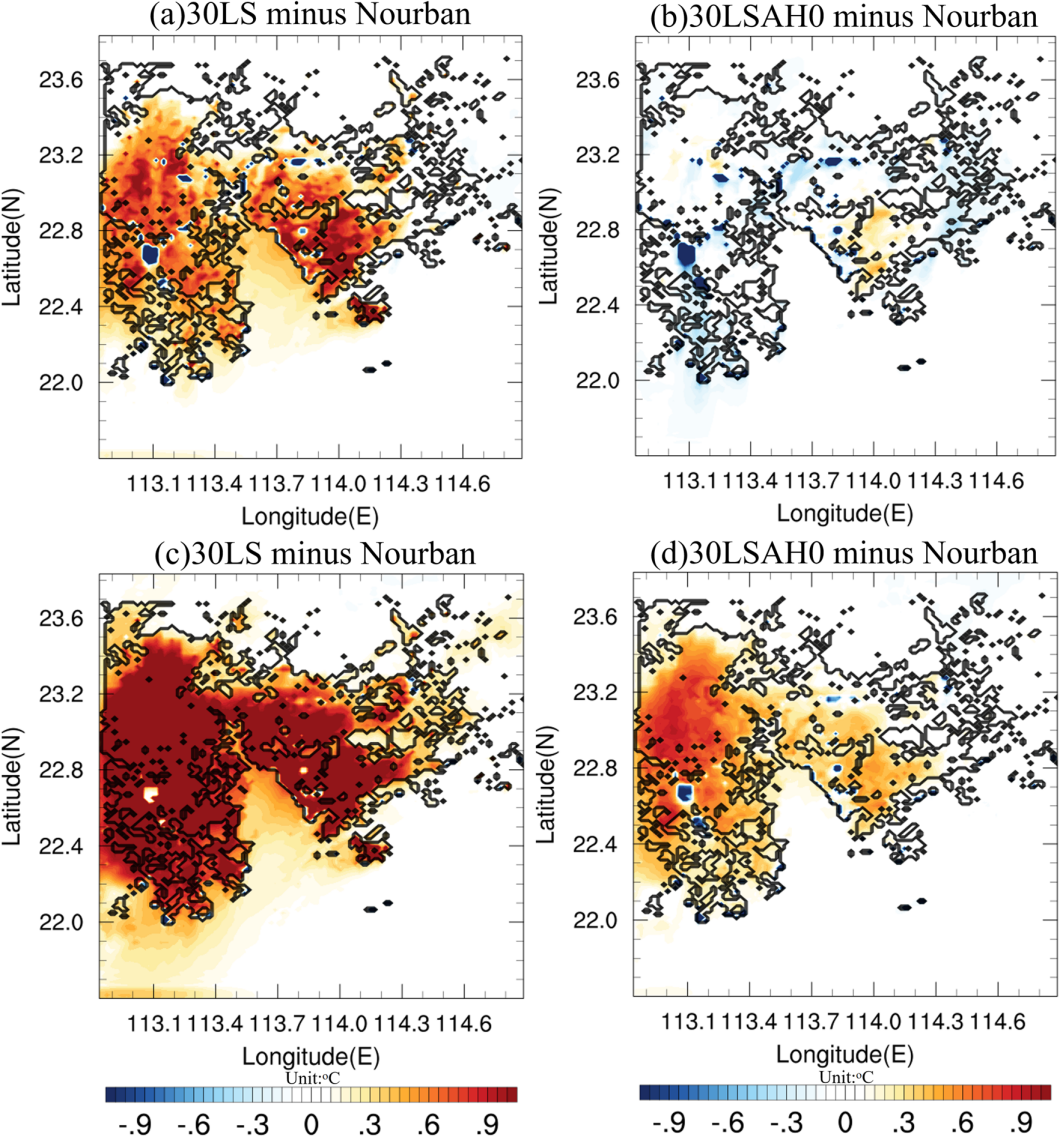
2-m temperature difference (units: °C) between (**a**) 30LS and Nourban and (**b**) 30LSAH0 and Nourban for Case 2013. Temperature is computed by averaging over rainfall period. See text for details. The urban boundaries are shown by black lines. (**c**,**d**) Same as (**a**,**b**) except for Case 2015. The maps were generated by the NCAR Command Language (NCL) Version 6.6.2^79^, http://dx.doi.org/10.5065/D6WD3XH5.

Figure 5 illustrate 2-m temperature differences between (a) 30LS and Nourban, (b) 30LSAH0 and Nourban for Case 2013, averaged over the rainfall period. The black contours delineate the boundary of the 2030 GBA mega-city. In Case 2013, urbanization leads to surface warming of approximately 0.5–1 °C within the GBA urban area, primarily in the northeastern and southwestern regions where major cities such as Guangzhou (113.29 E, 23.13 N), Shenzhen (114 E, 22.32 N), and Hong Kong (114.17 E, 22.3 N) are located. However, the warming induced by urbanization is minimal in the northern part of the city, particularly at the northern boundary of the GBA mega-cities, with temperature differences around 0–0.3 °C. Intriguingly, certain areas exhibit substantial temperature decreases due to urbanization, which can be attributed to the replacement of lakes in Nourban with low-intensity residential urban areas in 30LS, resulting in reduced surface thermal capacity. Furthermore, urbanization-induced temperature differences are also negligible in rural and marine areas, with a mere 0.2–0.3 °C of warming in the Greater Bay, downstream of the region associated with prevailing surface northerly winds. Additionally, the temperature changes caused solely by urban land use are shown in Fig. 5b, revealing minimal temperature differences across the entire GBA mega-city.

In contrast, Fig. 5 also present 2-m temperature differences for Case 2015 between (c) 30LS and Nourban, and (d) 30LSAH0 and Nourban. Case 2015 reveals a temperature increase of over 1 °C across almost the entire GBA urban area, with a warming of 0.3–0.5 °C in the downstream ocean area due to near-future urbanization. The temperature difference only remains weak at the city's northern boundary, and the temperature increase at the northern and eastern boundaries does not exceed 0.5 °C. Warming in the surrounding rural and marine areas due to urbanization is also negligible. In this case, the presence of urban land use also substantially increases 2-m temperatures in the entire western part of the urban area, as shown in Fig. 5d. Major GBA cities, such as Guangzhou and Foshan, experience temperature increases of about 0.4–0.8 °C, while most urban areas exhibit a temperature increase of approximately 0.3 °C due to urban land use alone.

**Figure 6:**
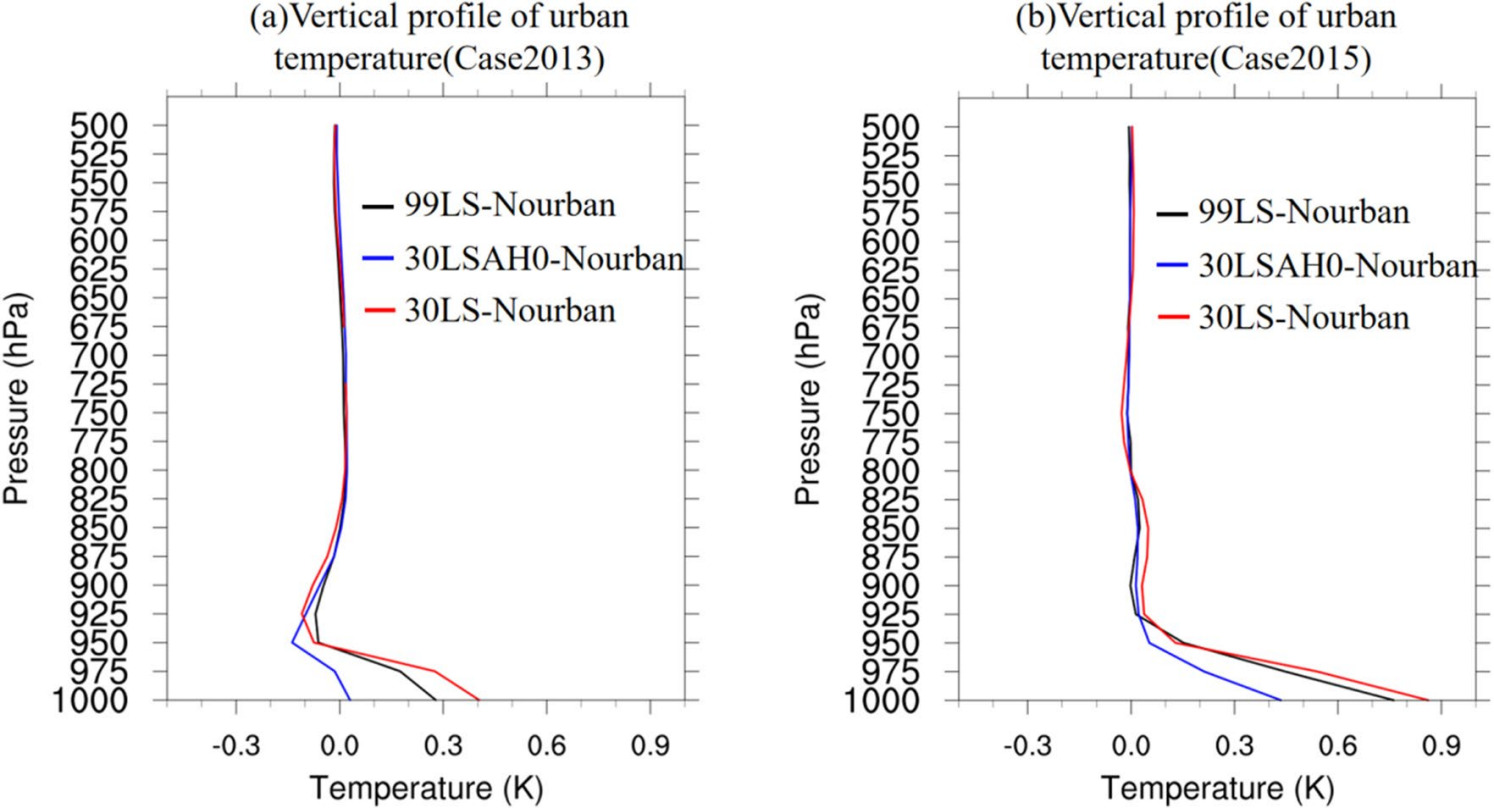
Vertical profile of urban temperature difference (units: K) averaged over the rainfall period for (**a**) Case 2013, and (**b**) Case 2015 from l000 to 500 hPa. Black, blue, and red lines represent, respectively, the temperature difference between 99LS and Nourban, 30LSAH0 and Nourban, and 30LS and Nourban.

To further examine temperature changes resulting from urbanization, Fig. 6 presents vertical profiles of urban temperature differences from 1000 to 500 hPa for (a) Case 2013 and (b) Case 2015, calculated by averaging temperature over the rainfall period. The black, blue, and red lines represent the temperature differences between 99LS and Nourban, 30LSAH0 and Nourban, and 30LS and Nourban, respectively. The most significant urban temperature differences are observed at the 1000 hPa level, with differences between 99LS, 30LSAH0, 30LS, and Nourban reaching 0.03 °C (0.43 °C), 0.28 K (0.75 °C), and 0.4 K (0.85 °C) for Case 2013 (Case 2015), respectively. In Case 2013, all temperature differences rapidly decrease with height, going from the 1000–925 hPa layer, with negative values above the 950 hPa level for all urban experiments compared to Nourban, which can be attributed to the strengthened frontal system and cold air intrusion induced by the UHI that not yet suppressed before rainfall period. The UHI is notably subdued even in 30LS, exhibiting a reduction of approximately 0.12 °C at 925 hPa in comparison to Nourban. The UHI then progressively increases to 0 above the 850 hPa level. Conversely, while the UHI also diminishes with height in Case 2015, temperature differences remain positive between the 1000 hPa and 800 hPa levels for all urban experiments compared to Nourban, and the UHI is nearly suppressed to 0 above the 800 hPa level.

In summary, although urbanization leads to increased urban surface temperatures in both Case 2013 and Case 2015, the warming is much more pronounced in Case 2015. The surface temperature difference attributable solely to urban land use is negligible in Case 2013, but still reach over 0.4 °C in Case 2015. Additionally, the UHI is considerably mitigated in Case 2013, which is associated with strong northerly winds and cold air intrusion. The distinct temperature differences between Case 2013 and Case 2015 suggest that the urbanization process and its impacts on local climate are sensitive to the specific meteorological conditions and synoptic backgrounds.

#### Precipitation

**Figure 7:**
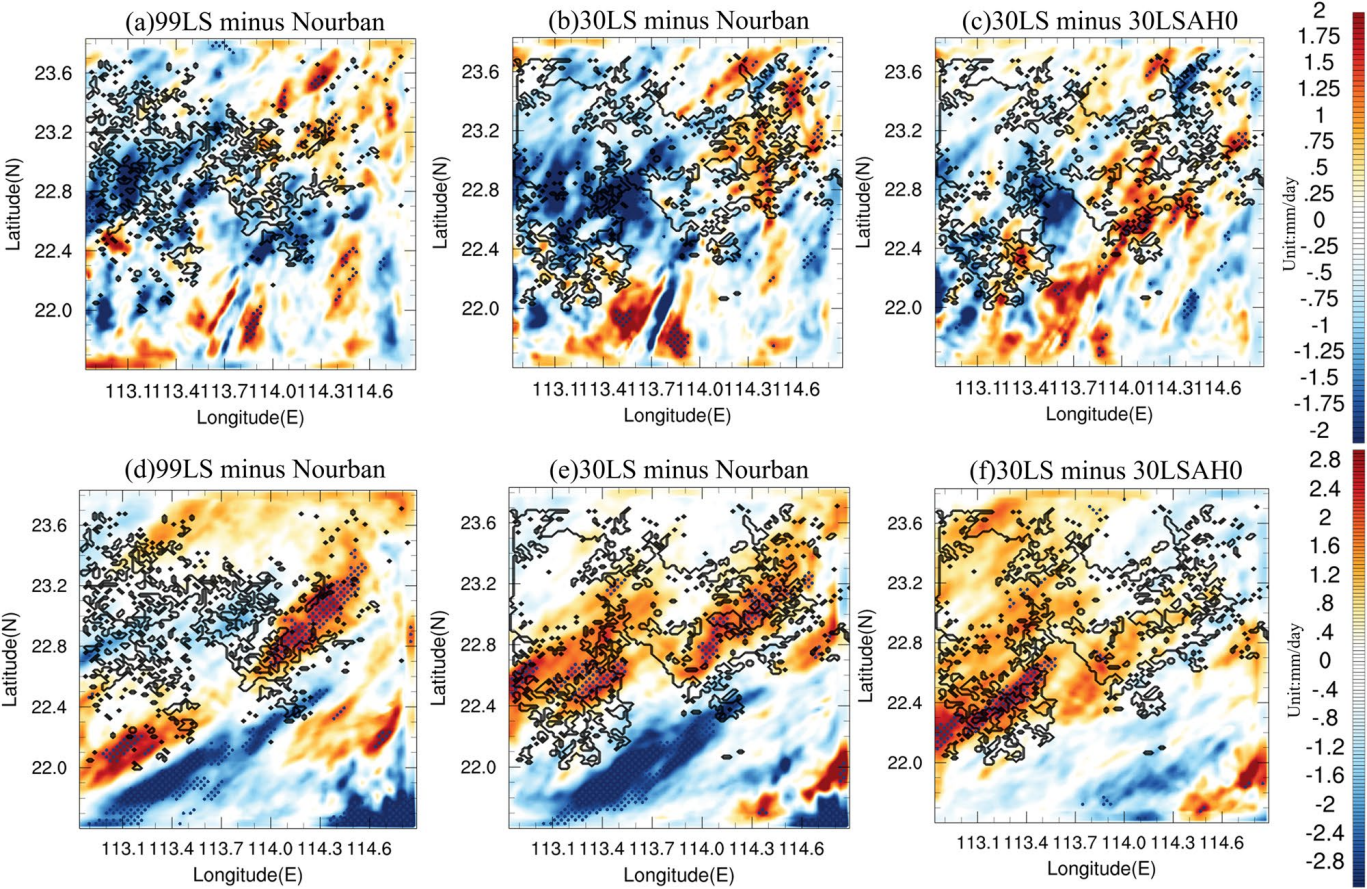
Accumulated rainfall (units: mm/day) difference between (**a**) 99LS and Nourban, (**b**) 30LS and Nourban, and (**c**) 30LS and 30LSAH0 for Case 2013. Black contours represent the boundary of the 1999 and 2030 GBA mega-city. Locations in which the difference exceeds the 95% significance level are denoted by black dots. (**d**–**f**) Same as (**a**–**c**) except for Case 2015. The maps were generated by the NCAR Command Language (NCL) Version 6.6.2^79^, http://dx.doi.org/10.5065/D6WD3XH5.

Figure 7 shows the accumulated rainfall difference between (a) 99LS and Nourban, (b) 30LS and Nourban, and (c) 30LS and 30LSAH0 for Case 2013, with black contours delineating the 2009 and 2030 GBA mega-city boundaries. Statistically significant differences (95% confidence level) are denoted by black dots. In urban areas, both 99LS and 30LS exhibit substantially lower precipitation intensities than Nourban, with the western and central parts of the city experiencing significant reductions in accumulated precipitation, exceeding − 2 mm/day. Precipitation variability in the surrounding rural and oceanic regions is highly irregular, but 30LS still exhibits slightly enhanced precipitation in the eastern part of the city. Furthermore, AH does not increase precipitation in urban areas (see Fig. 7c), and its influence on precipitation changes is weak and insignificant, with most urban areas experiencing less than 0.5 mm/day differences, which is consistent with the suppressed low-level UHI in Case 2013 (see Fig. 6a). Precipitation is enhanced only in oceanic areas downwind of the city, with differences reaching 1.5 mm/day.

Figure 7 also illustrates the accumulated rainfall differences for Case 2015 between (d) 99LS and Nourban, (e) 30LS and Nourban, and (f) 30LS and 30LSAH0. 99LS still exhibits slightly decreased precipitation in urban areas located between 22.6 and 23 N, while significantly enhanced rainfall is observed in the downstream area southeast of the city. This is consistent with literature suggesting that urbanization tends to enhance precipitation in downstream area^56–60^, and may be due to weak UHI intensity in 1999 GBA city^24,25^. In contrast, near-future urban development markedly intensifies precipitation in the central, eastern, and southern GBA mega-city (30LS minus Nourban, see Fig. 7e), with precipitation enhancement ranging from 1.5 to 2.5 mm/day in the southern and eastern urban areas compared to Nourban, and most enhanced precipitation areas pass the 95% significance threshold. However, rainfall changes are minimal in the northwestern part of the city. Furthermore, AH enhances precipitation across the entire urban region (see Fig. 7f), with increases varying from 1 to 2.5 mm/day. Despite this, signals over most areas do not pass the 95% significance level, and significant precipitation enhancement is only observed in the city's southwestern portion. In contrast, AH has a negligible effect on precipitation in the surrounding rural and marine areas. To better understand extreme precipitation enhancement during Case 2015, snapshots of hourly precipitation for (a) 99LS, (b) 30LS, (c) Nourban, and (d) 30LSAH0 during the hour of peak urban rainfall in Case 2015 are examined (see Fig. S2). In Nourban, precipitation covers the entire land area and part of the ocean within the innermost domain, with intensities ranging from approximately 4 to 12 mm/h. Heavy rainfall occurs in the southern GBA land area and the Greater Bay. Hourly peak rainfall intensity ranges from 4 to 8 mm/h in the northern and central GBA. Conversely, 99LS and 30LS exhibit enhanced rainfall across the entire GBA mega-city, with the central and eastern regions receiving increased rainfall from 4 to 8 mm/h in Nourban to 6–14 mm/h in 30LS. Compared to Nourban, 30LSAH0 also features stronger precipitation in the city's central region, but rainfall intensity diminishes in the southern portion and the ocean. AH also augments rainfall in most urban areas at the hour of peak urban rainfall.

**Figure 8:**
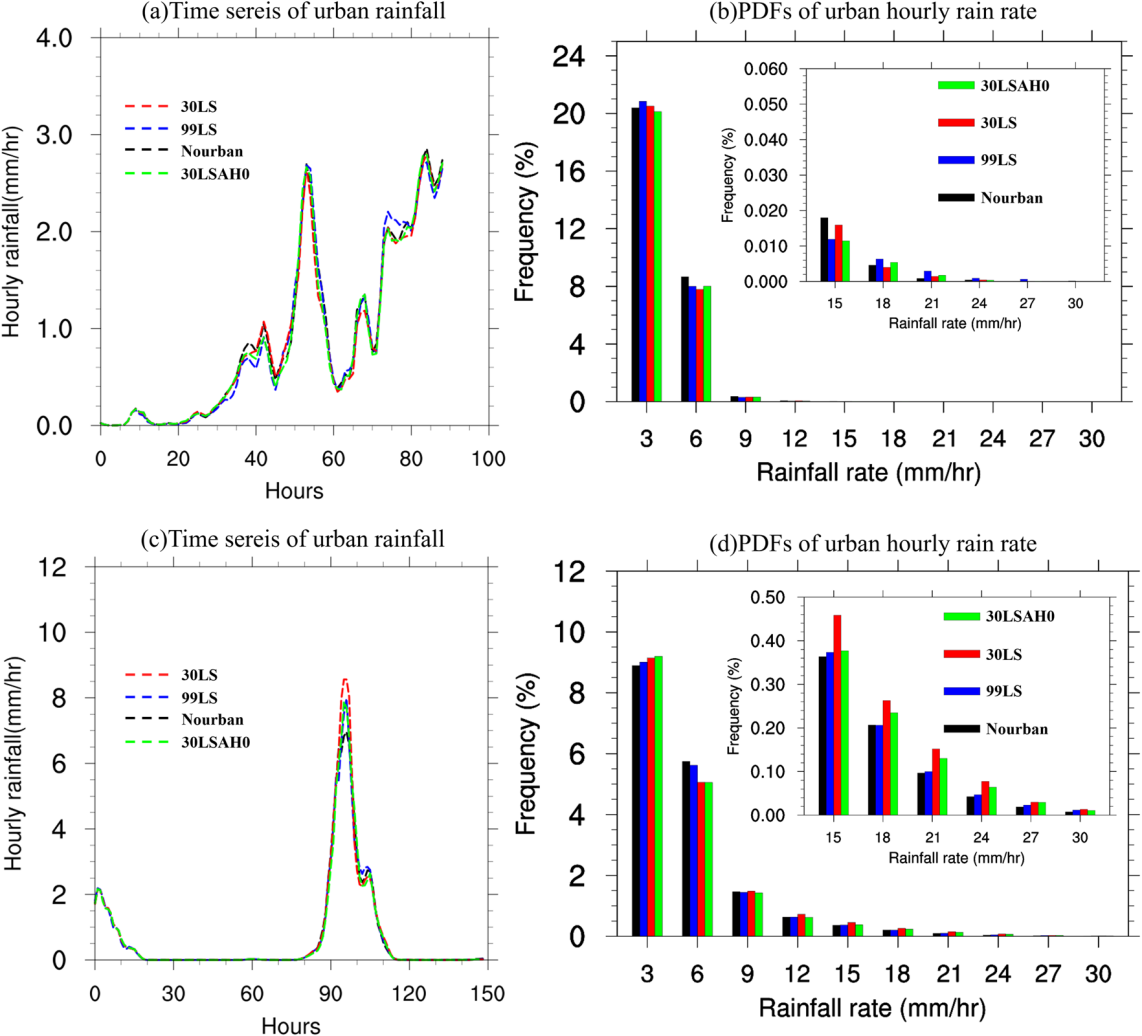
(**a**) Time series of hourly rainfall (units: mm/h) over urban locations within GBA area for Nourban (black), 99LS (blue), 30LSAH0 (green), and 30LS (red) for Case 2013 based on model simulation. (**b**) PDFs of hourly precipitation rates over urban locations within GBA, within the ranges of 1–3, 3–6, 6–9, 9–12, 12–15, 15–18, 18–21, 21–24, 24–27, and 27–30 mm/h for the Nourban (black), 99LS (blue), 30LSAH0 (green), and 30LS (red) experiments. (**c**,**d**) Same as (**a**,**b**) except for Case 2015.

Figure 8 examines the influence of urbanization on precipitation characteristics during Case 2013 by presenting (a) time series of urban rainfall and (b) PDFs of urban hourly rain rates for each experiment. Nourban, 99LS, 30LSAH0, and 30LS are represented by black, blue, green, and red lines (bars), respectively. In Case 2013, differences in urban hourly rainfall among experiments are minimal, not exceeding 0.1 mm/h during the initial peak rainfall period from t = 45 to t = 60 h. The hourly rainfall difference between 30 and 30LSAH0 is also negligible, suggesting that AH exerts a limited impact on urban hourly rainfall during Case 2013, consistent with the highly suppressed UHI (see Fig. 6a). PDFs of urban hourly rain rates reveal a mild urban effect on very light rainfall, but Nourban experiences more frequent rainfall than urban experiments (99LS, 30LSAH0, and 30LS) for rates between 3 and 15 mm/h. However, the decrease in rainfall probability due to urbanization is also modest, ranging from 5 to 30% reduction. In contrast, urban experiments exhibit higher probabilities of rainfall exceeding 20 mm/h, particularly 99LS, though the results are not robust due to the small absolute rainfall probabilities. As a result, the urban influence on winter precipitation during Case 2013 is weak and potentially negative for the GBA mega-city.

For Case 2015, Fig. 8 also presents analogous plots for (c) time series of urban rainfall and (d) PDFs of urban hourly rain rates. Urban rainfall commences at t = 70 h and peaks at t = 93 h. Peak precipitation intensities for Nourban, 99LS, 30LSAH0, and 30LS are 7 mm/h, 8 mm/h, 8 mm/h, and 8.7 mm/h, respectively, with the difference between 30LS and Nourban at peak hours being significant at the 95% confidence level according to the Student's t-test. Urban precipitation enhancement is robust only from t = 90 to t = 95 h. On the other hand, the PDFs of urban hourly rain rates reveal that near-future urbanization considerably increases rainfall frequency over the GBA mega-city (30LS minus Nourban), while the difference between 99LS and Nourban is insignificant. Comparing urban experiments (99LS, 30LSAH0, and 30LS) to Nourban, rainfall occurrence rates rise for hourly rainfall across all ranges (except 3–6 mm/h), particularly for rates exceeding 12 mm/h. For hourly rain rates between 10 and 30 mm/h, the probability increases markedly by 30–80% in 30LS compared to Nourban, but no more than 10% in 99LS compared to Nourban. This implies an increasing trend in the urban impact on GBA extreme winter precipitation in the near future compared to the historical era, as urban areas expand and upgrade. Furthermore, without AH in urban areas, 30LSAH0 exhibits decreased hourly rainfall probabilities across most ranges relative to 30LS, but it still outperforms Nourban in terms of rainfall frequency in almost all ranges. Consequently, near-future urbanization enhances both rainfall intensity and probability over the GBA mega-city during Case 2015. In summary, despite employing identical model configurations, the impact of urbanization on winter precipitation varies between cases. Results demonstrate that during Case 2013, urbanization exerts a weak influence on precipitation intensity and probability in cities, even producing a negative effect on hourly precipitation probability within the 3–20 mm/h range. In contrast, during Case 2015, near-future urbanization significantly intensifies both rainfall metrics, with the UHI effect primarily driving this enhancement. But the change of precipitation in 99LS is still negligible compared to Nourban.

#### Mechanism of urban impact on winter precipitation

To elucidate the varying urban precipitation changes under different synoptic backgrounds due to urbanization, we further investigated the mechanisms underlying urban influence on extreme winter precipitation. First, surface evaporation driven by urban land use is lower across the GBA mega-city than that associated with cropland land use (see Fig. S3). This reduction can be attributed to impermeable surfaces in urban areas, leading to decreased surface evaporation in these regions. As a consequence, a decline in water vapor is observed at the surface and in the lower atmospheric layers of urban areas.

**Figure 9:**
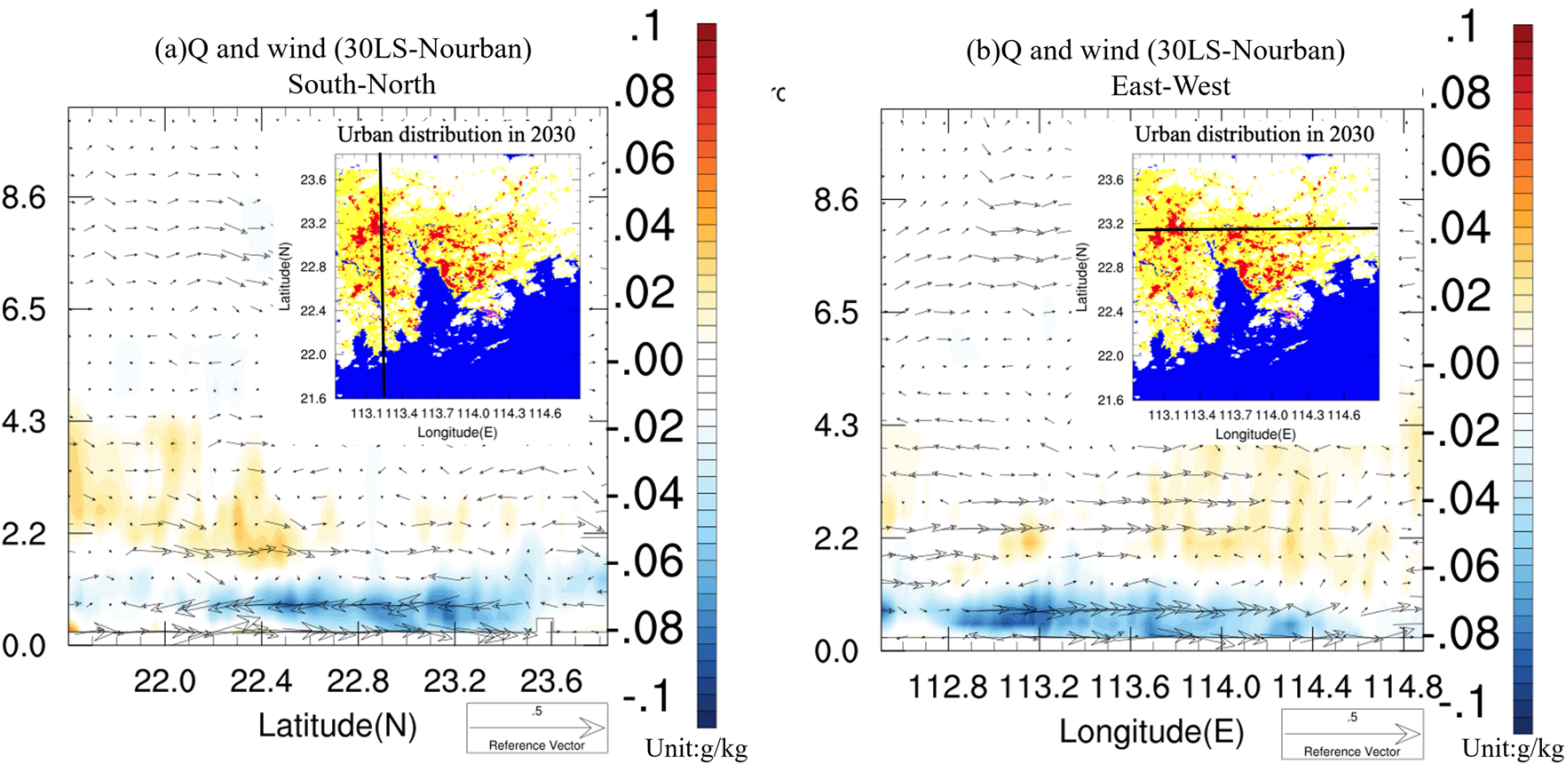
(**a**) North–south cross section of difference of hourly vertical wind vector (units: m/s) and specific humidity (units: g/kg), averaged over rainfall period of Case 2013, between 30LS and Nourban. Black line in inset represents the location of the cross-sectional plane. (**b**) Same as (**a**) except for the East–west cross section. The maps were generated by the NCAR Command Language (NCL) Version 6.6.2^79^, http://dx.doi.org/10.5065/D6WD3XH5.

Moreover, Fig. 9 presents the (a) south-to-north and (b) west-to-east cross-sections of vertical specific humidity and wind vector differences between 30LS and Nourban for Case 2013, with the black line in the inset figure indicating the cross-sectional plane. It is evident that specific humidity decreases markedly at the surface and low-level atmosphere due to urbanization, with reductions ranging from 0.04 to 0.08 g/kg in both directions. Conversely, enhanced southerly winds are observed above 2 km over the southwestern portion of the GBA, resulting in a slight increase in specific humidity. However, this enhancement is confined to the southwestern and eastern areas of the GBA and remains weak overall. The substantial reduction in water vapor content within the lower layers of the urban atmosphere, stemming from weakened surface evaporation, primarily accounts for the decreased precipitation in the GBA mega-city during Case 2013. Simultaneously, urban 2-m temperatures are considerably suppressed throughout rainfall period in Case 2013 as cold air dominates the entire synoptic condition, leading to a weakened UHI (see Fig. 5a,b). Although the UHI still contributes to slightly enhanced southerly winds and humidity above 2 km altitude in the southern part of the GBA mega-city, its impact is not comparable to the drying of the lower atmosphere induced by the urbanization-driven decrease in evapotranspiration and is insufficient to support urban precipitation.

**Figure 10:**
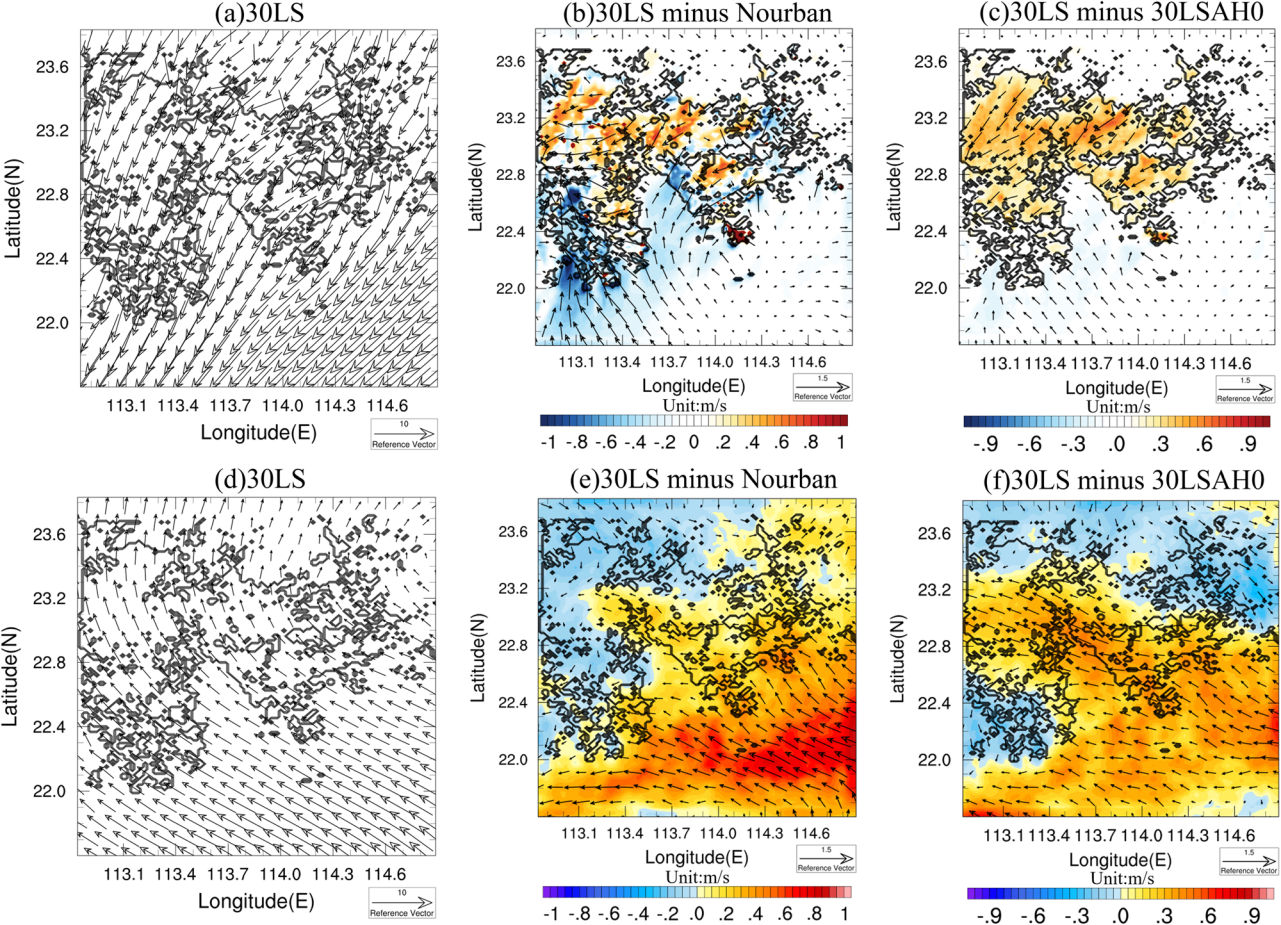
(**a**) Surface wind (unit: m/s) in 30LS, averaging over rainfall period of Case 2015, black contour represents the 2030 GBA mega-city. (**b**) Surface wind speed difference and wind difference between 30LS and Nourban, averaging over the rainfall period, with the wind speed difference and wind difference showing by shaded and vector. (**c**) Same as (**b**) except for difference between 30 and 30LSAH0. (**d**–**f**) Same as (**a**–**c**) except for 850 hPa wind. The maps were generated by the NCAR Command Language (NCL) Version 6.6.2^79^, http://dx.doi.org/10.5065/D6WD3XH5.

However, in Case 2015, the UHI persists, as shown in Figs. 5 and 6. Figure 10a gives the surface wind in 30LS, averaged over the rainfall period. During this period, prevailing surface northerly winds dominate the entire GBA, with wind speeds reaching 3–4 m/s on land and 5–6 m/s over the ocean. Figure 10 also presents the wind speed and wind vector differences between (b) 30LS and Nourban, and (c) 30LS and 30LSAH0 for Case 2015, with shaded areas representing wind speeds and vectors indicating wind direction. In comparison to Nourban, 30LS exhibits stronger northerly (northeasterly) winds in the northeastern (northwestern) part of the GBA mega-city, with wind speeds intensifying by approximately 0.3–0.5 m/s. Northerly winds are significantly weakened to the south of the city, and stronger southerly winds are observed over the ocean compared to Nourban. The influence of AH on surface wind is depicted in Fig. 10c (30LS minus 30LSAH0), demonstrating a considerable enhancement of northeasterly winds over the entire urban area and stronger southerly winds over the ocean. We propose that both the UHI effect and urban surface contribute to surface wind changes, with the former yielding a larger (smaller) temperature gradient between urban and northern rural areas (ocean areas), thereby accelerating (decelerating) the prevailing surface northerly winds over the northern part of the urban area (southern part of the urban area and ocean area). The latter results in reduced wind speeds in urban areas due to increased urban surface roughness. By altering circulation, urban influence also promotes greater moisture flux convergence and convection in urban areas, ultimately enhancing precipitation.

Regarding low-level wind, Fig. 10 also displays the 850 hPa wind speed and wind vector in 30LS, averaged over the rainfall period. In contrast to surface winds, the 850 hPa winds shift from northerly to southeasterly and southerly over the GBA. The differences in wind speed and wind vector between (e) 30LS and Nourban, and (f) 30LS and 30LSAH0 for Case 2015 are also depicted. Urbanization is shown to lead to significantly stronger southeasterly winds over the ocean and the southeastern portion of the GBA mega-city, which can further increase water vapor supply from the ocean to urban areas. Conversely, when considering only the impact of AH (30LS minus 30LSAH0), enhanced southeasterly winds are observed over the ocean and nearly the entire GBA mega-city compared to Nourban, which can also result in a more humid atmosphere and support rainfall in urban areas.

**Figure 11:**
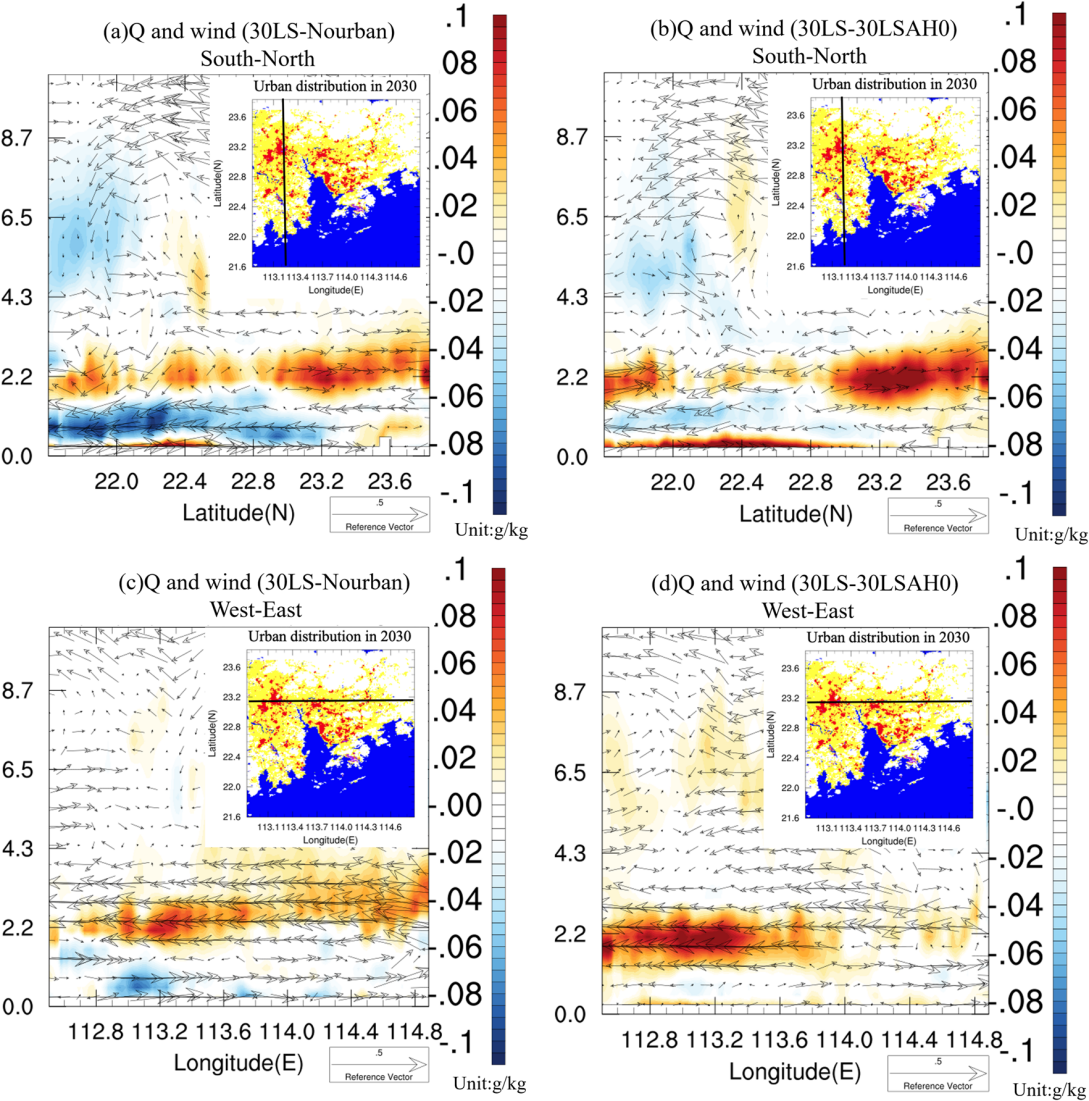
(**a**) South-to-North cross section of difference of hourly vertical wind vector (units: m/s) and specific humidity (units: g/kg), averaged over rainfall period of Case 2015, between 30LS and Nourban. Black line in the inset represents the location of the cross-sectional plane. (**b**) Same as (**a**) except for difference between 30 and 30LSAH0. (**c**,**d**) Same as (**a**,**b**) except for West-to-East cross sectional plane. The maps were generated by the NCAR Command Language (NCL) Version 6.6.2^79^, http://dx.doi.org/10.5065/D6WD3XH5.

In order to further explore the urban impacts on local circulation and humidity during Case 2015, Fig. 11 presents the south-to-north cross-section of vertical specific humidity and wind vector differences averaged over the rainfall period between (a) 30LS and Nourban, and (b) 30LS and 30LSAH0. The inset figure's black line represents the cross-section plane. Notably, specific humidity below 1.5 km height decreases in 30LS due to reduced urban surface evaporation and the strengthening of synoptic northerly winds. However, increased humidity is observed at near-surface levels from 22 to 22.6 N, attributable to the strengthening of southerly winds south of the GBA mega-city caused by the UHI. Furthermore, elevated water vapor content is found above 1.5 km throughout the urban area, particularly from 22.8 to 24 N, with a specific humidity increase of approximately 0.04 to over 0.1 g/kg compared to Nourban. This enhanced humidity is associated with intensified low-level southerlies supplying water vapor to urban areas from the ocean. Considering only the impact of AH (see Fig. 11b), the enhancement of specific humidity at near-surface levels and above 1.5 km becomes more pronounced than in Fig. 11a, while the drier airflow caused by stronger northerly winds between 500 m and 1.5 km altitude weakens.

Cross-sections of specific humidity and wind in other directions are also examined. The west-to-east cross-section of vertical specific humidity and wind vector differences averaged over the rainfall period is also shown in Fig. 11 between (c) 30LS and Nourban, and (d) 30LS and 30LSAH0. Compared to Nourban, the specific humidity slightly decreases at low levels in 30LS, but a significant enhancement of water vapor content is observed above the height of 2 km, with an increase of approximately 0.4–1 g/kg spanning almost the entire area. Concurrently, stronger easterly winds are detected at altitudes of 2–4.5 km in 30LS compared to Nourban, aligning with the enhanced low-level southeasterly winds over the ocean and urban area (see Fig. 10e). When comparing 30LS and 30LSAH0, it can be inferred that AH results in a dramatic increase in specific humidity and intensified easterly winds in the western part of the GBA mega-city, where is a highly urbanized area and receives more precipitation due to AH. Additionally, a slight enhancement of specific humidity near the surface in 30LS compared to 30LSAH0 can be attributed to increased wind convergence due to AH.

Finally, Table 1 shows the urban moisture flux divergence difference integrated over all layers for Case 2013 and Case 2015, averaged over the rainfall period. Differences between 30LS and Nourban, 99LS and Nourban, and 30LSAH0 and Nourban are displayed, with results further categorized into thermodynamic (dq) and dynamic (duv) terms. Upon comparing 30LS and Nourban, urban moisture flux convergence is found to be higher in 30LS for both Case 2013 and Case 2015, but the difference is substantially larger in Case 2015, reaching − 0.01525 g/m^2^/s compared to only − 0.000389 g/m^2^/s in Case 2013 (negative difference signifies more moisture flux convergence). Note that this variation in moisture flux convergence is primarily induced by the dynamic term. The difference in moisture flux convergence between 99LS and Nourban is only half of that between 30LS and Nourban in Case 2015. The enhancement is also notably stronger during Case 2015 than during Case 2013. Furthermore, considering the impact of urban land use, results indicate that there is an even larger urban moisture flux divergence in 30LSAH0 compared to Nourban for both Case 2013 and Case 2015, which can be attributed to contributions from both thermodynamic (dq) and dynamic (duv) terms. Consequently, urbanization substantially enhances moisture flux convergence in urban areas during Case 2015, with larger urban convergence being observed in 30LS, while the convergence difference between 99LS and Nourban is relatively weaker. This can be primarily attributed to the UHI effect. However, this effect is negligible and considerably weaker during Case 2013. The presence of urban land use results in stronger moisture flux divergence in the GBA mega-city during the rainfall period of both cases.

**Table 1:** Vertically integrated moisture flux divergence difference between 99LS and Nourban, 30LSAH0 and Nourban, and 30LS and Nourban for each case.

Moisture flux divergence difference	Case 2013 (g/m^2^/s)	Case 2015 (g/m^2^/s)
30LS-Nourban	− 0.00039	− 0.01526
30LS-Nourban (dq term)	− 0.00144	− 0.00221
30LS-Nourban (duv term)	0.00107	− 0.01306
99LS-Nourban	− 0.0007	− 0.00837
99LS-Nourban (dq term)	0.00019	− 0.00149
99LS-Nourban (duv term)	− 0.00089	− 0.00706
30LSAH0-Nourban	0.04116	0.02187
30LSAH0-Nourban (dq term)	0.01753	0.01529
30LSAH0-Nourban (duv term)	0.02326	0.00658

Values are calculated by averaging over the GBA urban area and all ensemble members, with the duv (dq) term representing the dynamic (thermal dynamic) term.

## Summary and discussion

In this comprehensive study, we delved into the intricate relationship between urbanization and extreme winter precipitation within the GBA mega-city. Our investigation drew from observations and employed the WRF-SLUCM model to shed light on this complex relationship. We designed parallel experiments for model simulations, manipulating factors such as land use types (including urban land use in 1999 and projected 2030 scenarios, as well as cropland) and surface AH flux values.

Our observational results underscored the persistence of the UHI effect during winter, with temperature differences exceeding 1 °C between urban and rural areas. We noticed a reduction in the 99th percentile hourly rainfall in urban regions compared to their rural counterparts. This decrease was attributed to the drier atmosphere induced by lower evaporation and humidity from urban land surfaces. However, when examining extreme rainfall events such as annual maximum and the 99.99th percentile hourly rainfall during winter, urban areas experienced higher intensities than rural locales.

Our study delved further by comparing 11 cases with strong UHI effects and 12 cases with weak UHI cases. The findings highlighted the significant enhancement of winter extreme rainfall intensity and probability in urban areas, especially for rainfall exceeding 10 mm/h, when the UHI effect was pronounced.

We further examined two specific extreme events, namely Case 2013 and Case 2015, dynamically downscaling them over the GBA under various experimental scenarios. The numerical modeling outcomes showcased the high sensitivity of urban impacts on extreme winter rainfall to synoptic background conditions. In the case of 2013, urbanization had a minimal impact on precipitation and even led to a decrease in accumulated rainfall over the GBA mega-city. This reduction was attributed to decreased evaporation and atmosphere humidity due to urban surfaces, and the suppression of the UHI by northerly winds and cold air intrusion. Conversely, during Case 2015, the persistent UHI effect significantly amplified precipitation intensity and probability over the GBA mega-city in 30LS compared to Nourban, primarily due to the AH effect. However, the changes in precipitation in 99LS were relatively weak and insignificant. Near-future urban development-induced changes in circulation led to increased low-level water vapor over urban areas, along with enhanced urban moisture flux convergence compared to the Nourban scenarios, consistent with enhanced precipitation in the city during Case 2015.

In summary, our findings unvealed the intricate interplay between urbanization and winter extreme precipitation in the GBA mega-city. Although lower evapotranspiration from urban surfaces tends to create a drier low-level atmosphere (hence less urban precipitation), persistent UHI effects can lead to more intense wintertime extreme rainfall, especially in the 2030 urbanized scenario. This is mainly due to enhanced circulation and convection, facilitating the transport of additional water vapor from the ocean to coastal urban areas.

Our research addresses a critical knowledge gap regarding winter extreme precipitation and urban climate in the GBA. It highlights that urban impacts on extreme rainfall vary depending on different synoptic backgrounds, with the potential for positive urban effects on extreme winter rainfall. These insights have practical implications for future planning and policymaking in agriculture and transportation. However, we also recognize certain limitations in our methodology. Particularly, the UHI and the area of precipitation not only depends on human activity but also physical processes. The definition of UHI, based on seven urban and seven rural stations selected, is somewhat subjective in our observation analysis. Nevertheless, this approach provides valuable initial evidence and aids in forming hypotheses about the potential relationship between urban–rural temperature differences and extreme precipitation. Additionally, our simulation approach was somewhat simplified, categorizing urban areas within GBA mega-city as three land use types for SLUCM. Our future research endeavors will expand beyond the GBA to other mega-city clusters, such as the Yangtze River Delta (YRD) and the Beijing-Tianjin-Hebei area. Given their distinct geographical locations and synoptic backgrounds, we anticipate uncovering further nuances in urban impacts on extreme rainfall under various winter weather systems. To bolster our findings, we plan to simulate additional winter cases and employ a multi-layer urban canopy model (MLUCM) with 10 types of historical and future urban land use types to provide more detailed insights into the urban surface and UHI effects on winter rainfall.

## Methodology

### Observations and winter case study

**Figure 12:**
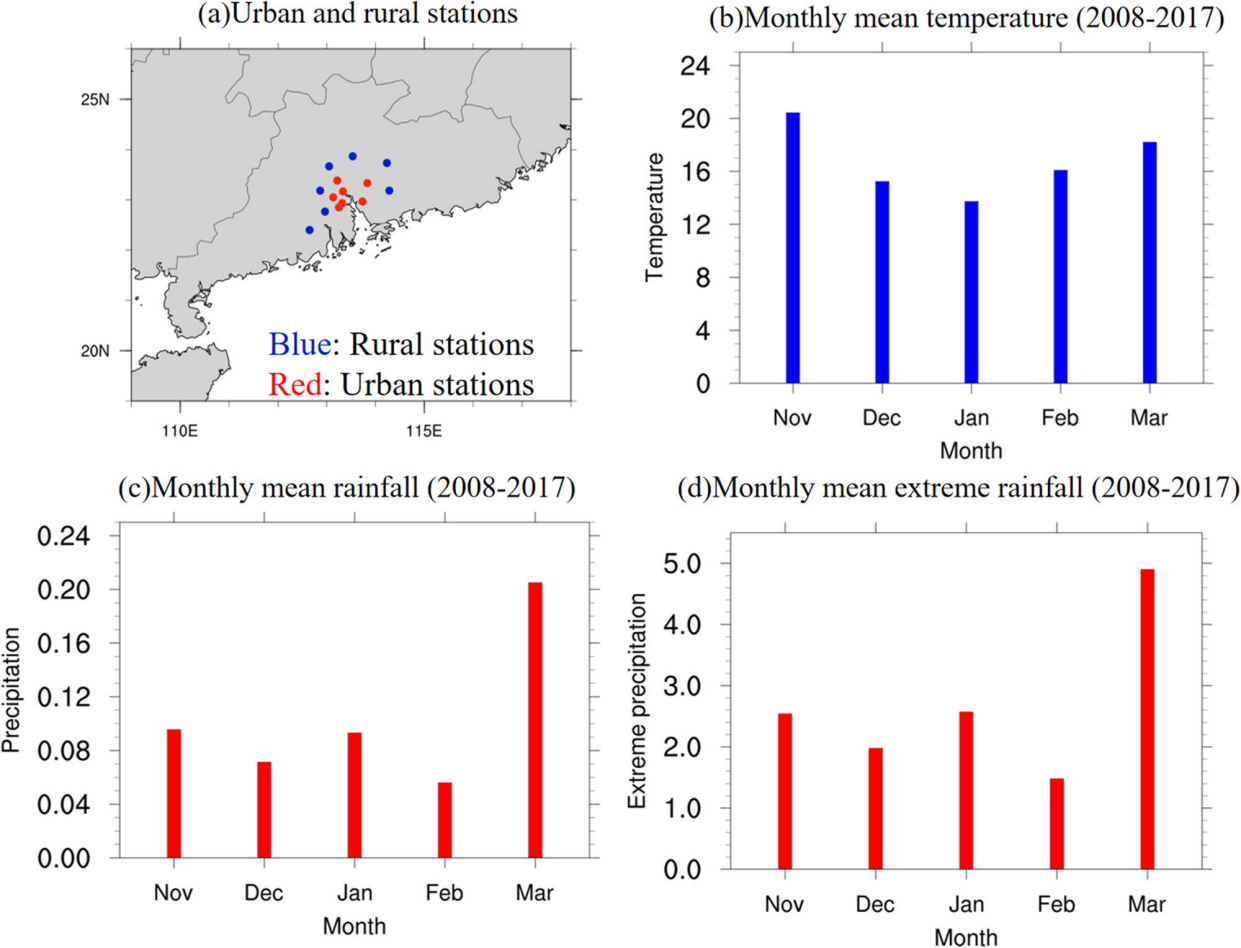
(**a**) Location of observation stations over South China, with red (blue) dots representing seven urban (rural) stations. Monthly mean (**b**) temperature (units: °C), (**c**) accumulated rainfall (units: mm/h), and (**d**) extreme rainfall (99th percentile of hourly rainfall, units: mm/h) in November, December, January, February, and March averaged from 2008 to 2017 for all urban and rural stations. The maps were generated by the NCAR Command Language (NCL) Version 6.6.2^79^, http://dx.doi.org/10.5065/D6WD3XH5.

This study aims to examine the urban influence on winter precipitation in GBA, using observational data from the China Meteorological Administration (CMA), consisting of 86 stations across SC with hourly data from January 2008 to December 2017. The dataset encompasses hourly measurements of precipitation, surface temperature, surface relative humidity, surface pressure, and surface U and V wind components. Within the GBA, seven urban and seven rural stations were further selected for analysis, the stations in SC that were not part of this selection were referred to as “other stations”. The locations of urban (red), rural (blue) stations are given in Fig. 12a. To define the winter period for this study, both temperature and precipitation in GBA were considered. Figure 12 also presents the monthly mean (b) temperature, (c) precipitation, and (d) extreme precipitation (99th percentile of hourly precipitation) for the 14 selected stations from 2008 to 2017. Near-surface temperatures at each station were standardized by recalculating them to sea-level values, using a temperature lapse rate of 0.6 °C/100 m. The 10-year average temperature exhibits a decrease from November to January, reaching the lowest monthly average of approximately 14.5°C in January, and increasing to around 18 °C in March. Meanwhile, the monthly mean accumulated rainfall (extreme rainfall) intensity from November to March ranges from 0.095 mm/h (2.5 mm/h) to 0.2 mm/h (5 mm/h). Similar intensities are observed for accumulated and extreme rainfall from November to February, with values not exceeding 0.1 mm/h and 3 mm/h. However, in March, these values reach 0.2 mm/h and 5 mm/h, showing a distinct difference from the other four months. Based on these findings, the extended winter season is defined as November through February (NDJF) in this study.

Moreover, 35 extreme rainfall events were identified by applying the threshold of the 99th percentile of hourly extreme rainfall, averaged across the 14 selected stations (see Fig. 12a). To further investigate the impact of UHI on extreme winter rainfall, these events were further categorized into strong UHI cases and weak UHI cases. This classification was determined based on the temperature difference between seven urban and rural stations, averaged from 1 day prior to the hour of rainfall peak until the peak hour of all 14 stations. A total of 11 strong UHI cases (urban–rural temperature difference > 0.8 °C) and 12 weak UHI cases (urban–rural temperature difference < 0.3 °C) were selected for further analysis. By examining these two distinct categories, the study aims to elucidate the role of the UHI effect on extreme rainfall events and provide insights into how urbanization may modulate winter precipitation in GBA mega-city.

### WRF model configuration and two winter cases

In order to assess the sensitivity of extreme winter precipitation to urbanization, the WRF model with the Advanced Research WRF (ARW) dynamical core version 4.3.3^80^ was employed to dynamically downscale two winter extreme rainfall cases, using boundary conditions from ERA5^61^. Nested domains for the downscaling experiments encompass East Asia/western North Pacific (2.23–43.82° N, 70.81–147.05° E, at 50 km × 50 km grid spacing), South China (19.94–27.09° N, 110.68–117.60° E, at 10 km × 10 km grid spacing) and GBA (21.5–23.83° N, 112.51–115.04° E, at 2 km × 2 km grid spacing), utilizing one-way nesting (see Fig. S4). The model extends up to a height of approximately 10 hPa. The physical parameterizations in the WRF model include the Noah Land Surface Model (LSM) to support the single layer urban canopy model (SLUCM) within the model environment^62,63^, the Rapid Radiative Transfer Model for General Circulation Model (RRTMG) for longwave radiation^64^, Dudhia’s short wave radiation scheme^65^, the WRF single-moment 6-class microphysics scheme^66^, Eta similarity theory for surface layer options^67^, the Bougeault-Lacarrere planetary boundary layer scheme^68^, and the simplified Arakawa-Schubert (SAS) GFS cumulus parameterization^69^ (for the outermost domain only). To ensure that WRF can reproduce the same synoptic-scale circulation as in the driving data, spectral nudging was applied in the outermost domain for U and V wind above 500 hPa, at the wavelength of about 1300 km, which is commonly used in simulation^70,71^.

In addition, dynamic downscaling was conducted for two winter extreme precipitation cases, namely Case 2013 (2013-12-13 to 2013-12-17) and Case 2015 (2015-12-05 to 2015-12-11), with a 2 km × 2 km grid spacing. For each case, we executed ensemble integration with nine members at 1-h intervals for the starting simulation time and averaged the results across all ensemble members.

### UCM, model experiments, and local climate zone (LCZ) data

To assess the impact on urban development on extreme winter rainfall, four sets of experiments were conducted using the WRF model with varying land use configurations in the innermost domain. In the Nourban experiment, the GBA urban land use was replaced by cropland; in the 99LS and 30LS experiments, urban land use information from 1999 and projected for 2030^72^ in the GBA area was incorporated into the SLUCM; and in the 30LS-AH0 experiment, 2030 land use data with zero AH in the urban area was employed for dynamical downscaling. For the highly urbanized GBA region, detailed land use/land cover change (LULCC) data for 1999 and a near-future projection for 2030 were developed based on the World Urban Database and Access Portal Tools (WUDAPT) protocol^73,74^. These datasets had a spatial resolution of 100 m × 100 m and were classified according to the local climate zone (LCZ) scheme^75^. The LCZ classification encompasses ten urban land use types including Compact High-Rise, Compact Mid-Rise, Compact Low-Rise, Open High-Rise, Open Mid-Rise, Open Low-Rise, Lightweight Low-Rise, Large Low-Rise, Sparsely Built, and Heavy Industry^75^. Each LCZ type is associated with a unique set of UCM parameters and attributes^76^, such as surface albedo, building height, and urban ratio. These parameters were specifically tailored for the GBA mega-city^13,32^. Due to the lack of data, we prescribed the same urban land use information for the GBA in 1999 (the historical era) and 2030 (the projected near-future era)^32^.

2030 near-future urban LCZ maps were developed by Huang et al. (2021). These projections utilized the Geographical Simulation and Optimization System (GeoSOS)—Future Land Use Simulation (FLUS) model^77,78^ based on historical LCZ maps and the interactions between different land uses. The process involved three main steps:

- An Artificial Neural Network (ANN) was applied to learn the occurrence probability of each land use from historical LCZ maps, taking into account geographical factors such as slope, aspect, elevation, and distances to city centers, roads, and highways, as derived from current LCZ maps.
- Future demands for urban land use and land cover changes were predicted based on demographic and socioeconomic development trajectories.
- A Cellular Automata (CA) model was employed to simulate land use conversions using the current LCZ maps (2014 LCZ maps), with occurrence and neighborhood influence probabilities iterated until future demands were met. The CA model then generated the projected near-future maps.

To reduce complexity and simulation uncertainties, the ten urban LCZ types were consolidated into three categories: “Low Intensity Residence” (type 1) comprises Open Mid Rise, Open Low Rise, Sparsely Built, Open High Rise, Lightweight Low Rise, Large Low Rise; “High Intensity Residence” (type 2) comprise Compact Mid Rise; Compact Low Rise, and finally, “Commercial and Industrial” (type 3) includes both Compact High Rise and Heavy Industry.This regrouping was based on the AH values of each urban LCZ, with higher AH values corresponding to more intensive land use types, e.g., Compact High Rise and Heavy Industry have the highest AH, which exceed 150 W/m^2^, are regrouped as “Commercial and Industrial”. Urban LCZs with AH lower than 25 W/m^2^ are regrouped as “Low Intensity Residence”^32^. Table 2 presents the UCM parameters prescribed for these three land use types, with values derived from the WUDAPT project for GBA-based parameters and recalculated based on the new classification. Also shown in Fig. S4 are the 1999 and 2030 urban land use distributions (after regrouping) in the innermost model domain, with yellow, red, and purple indicating Low Intensity Residence, High Intensity Residence, and Commercial and Industrial, respectively.

**Table 2:** Values of UCM parameters prescribed for three types of urban categories.

UCM parameter	Type 1	Type 2	Type 3
Anthropogenic heat [W m^−2^]	15.0	50.0	200.0
Building height [m]	13.31	14.01	53.92
Urban fraction	0.4	0.9	0.7
Standard deviation of roof height [m]	10.42	5.64	33.18
Roof width [m]	38.01	14.28	35.5
Road width [m]	22.98	13.91	29.6
Surface albedo of road	0.2	0.17	0.18
Surface albedo of roof	0.142	0.165	0.115
Surface albedo of building wall	0.208	0.2	0.22

### Model evaluation

**Figure 13:**
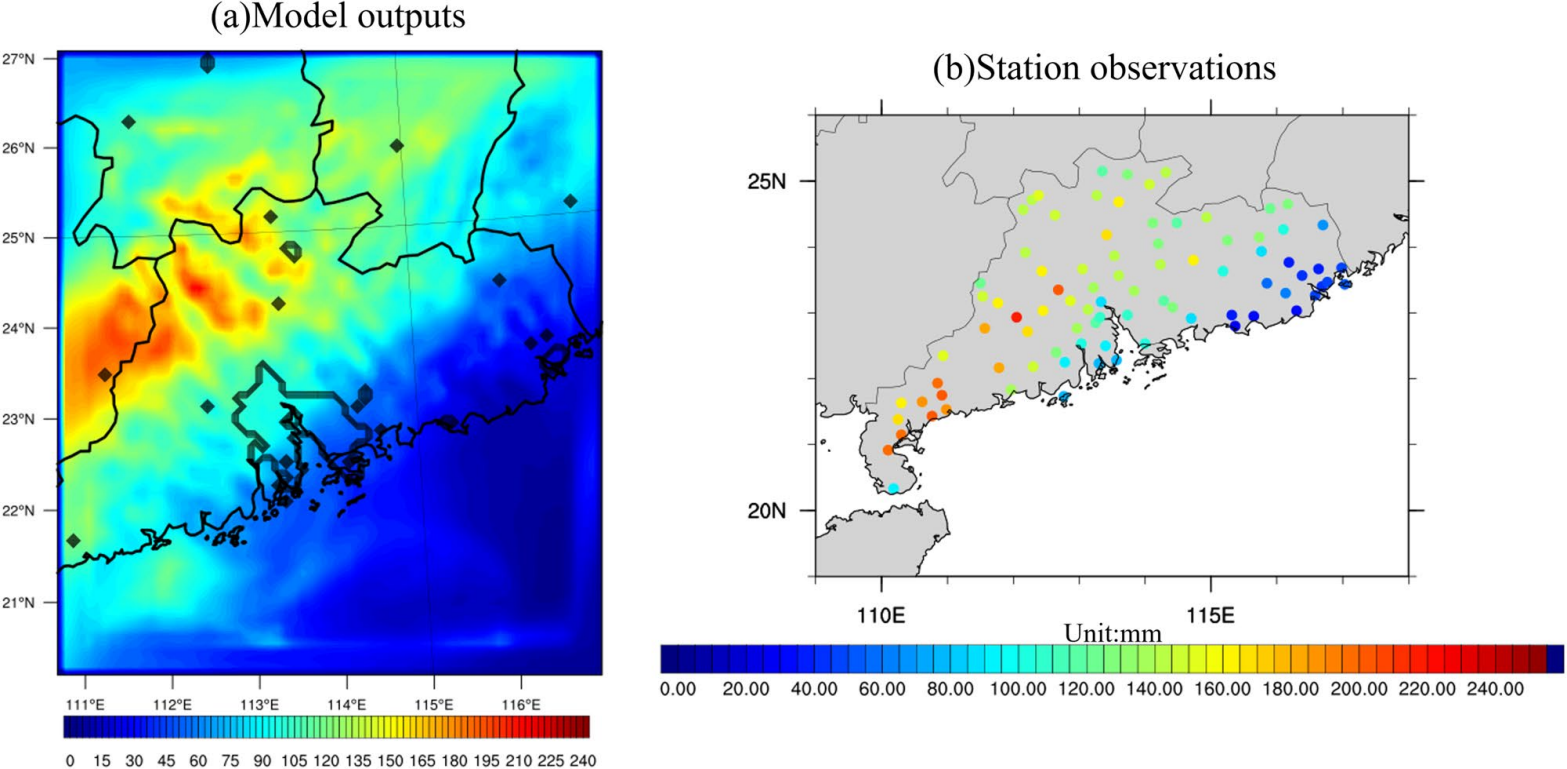
(**a**) Accumulated rainfall (units: mm) averaged over Case 2013 from model outputs. Black contours represent the GBA mega-city and boundary of province. (**b**) Similar to (**a**) except for station observations. The maps were generated by the NCAR Command Language (NCL) Version 6.6.2^79^, http://dx.doi.org/10.5065/D6WD3XH5.

To evaluate the performance of the WRF model in simulating these two specific winter extreme rainfall events, weather parameters from the model outputs were compared with both station observations and ERA5 reanalysis data. Figure 13 illustrates the accumulated rainfall derived from (a) model outputs, and (b) station observations during Case 2013. Black contours delineate boundaries of provinces and the GBA mega-city. Observations reveal a prominent rainfall pattern associated with the cold frontal system, primarily manifesting in the northwestern and southwestern part of SC. The rainfall amount exhibits a gradual decrease from west to east, with accumulated precipitation reaching approximately 180 to over 220 mm in the western part of SC, in stark contrast to the eastern part of SC, where the values do not exceed 20 mm. Concurrently, the GBA mega-city records precipitation amounts ranging from 80 to 160 mm for Case 2013. The model's performance aligns satisfactory with observations over most of the SC area, particularly in the northern and eastern parts. The model effectively captures the west-to-east decreasing precipitation gradient observed across SC. However, the model noticeably underestimates rainfall in the southwestern part of SC, likely due to its proximity to the edge of the model's domain. Meanwhile, a slight overestimation of rainfall is observed in the northern region of SC. The model's simulation of rainfall intensity over the GBA urban area is highly consistent with the observations, albeit it marginally underestimates the intensity over the southeastern and coastline of the GBA.

For Case 2015, Fig. 14 gives the accumulated rainfall derived from (a) model output, and (b) station observations. Observations indicate an accumulated rainfall of about 140–180 mm in the eastern and northeastern part of SC, with a decreasing pattern from northeast to southwest. Around 100–130 mm of total precipitation is observed in the GBA mega-city. On the other hand, the model results tend to overestimate the rainfall intensity in the GBA mega-city and part of southwestern SC, with model cumulative rainfall reaching 150 mm in the GBA mega-city, which overstates the rainfall by about 20–30 mm. However, the simulated precipitation aligns reasonably with observations over the northern and eastern parts of SC. The presence of strong rainfall over the GBA, despite slight overestimation, is deemed acceptable and supportive of our study's objectives. The subtle deviations from observed data do not undermine the overall validity of the model, reinforcing its utility as an effective tool in simulating and understanding extreme rainfall events.

**Figure 14:**
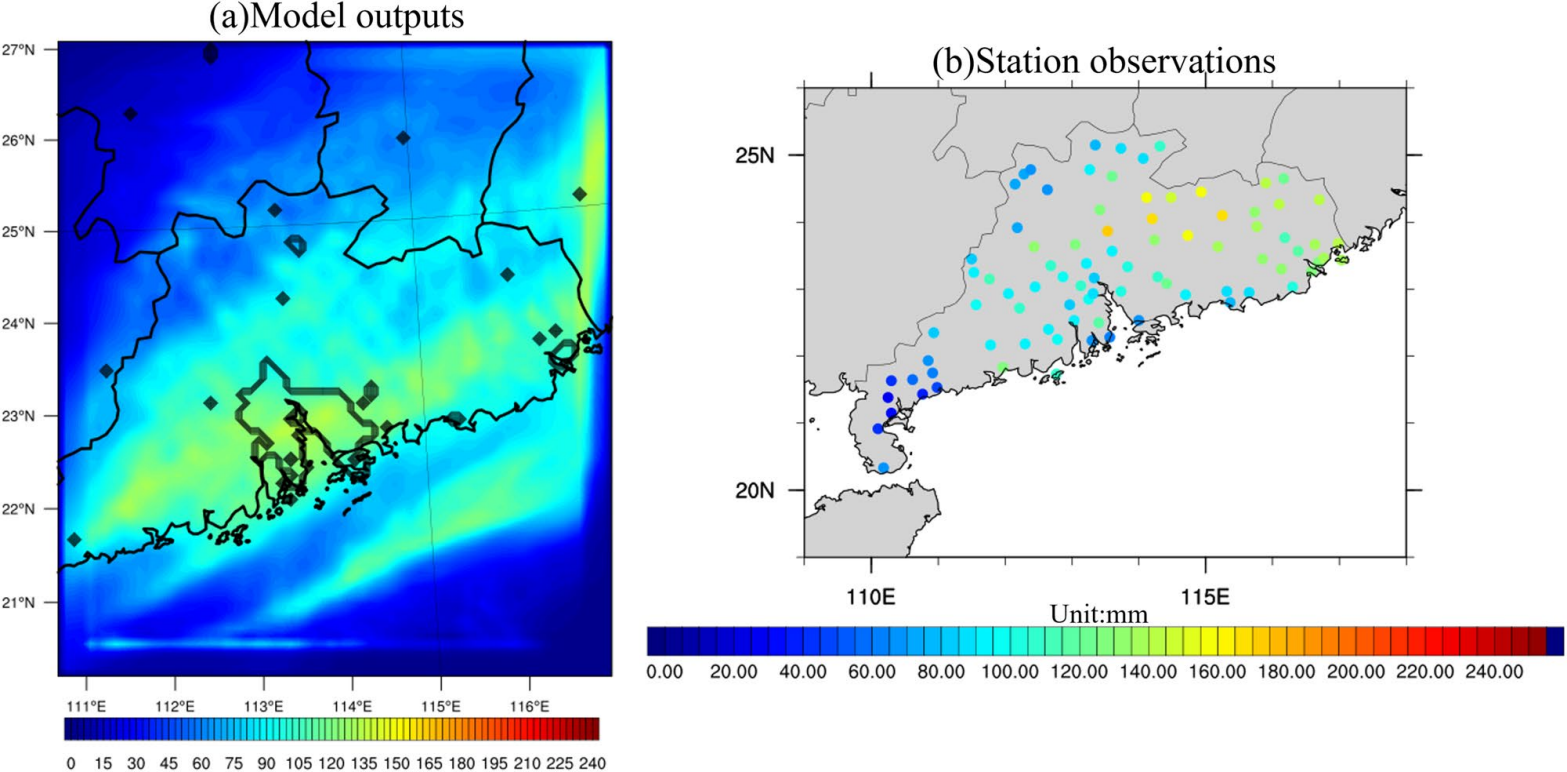
Same as Fig. 13 except for Case 2015. The maps were generated by the NCAR Command Language (NCL) Version 6.6.2^79^, http://dx.doi.org/10.5065/D6WD3XH5.

Moreover, evaluation of the model's performance in simulating temperature and circulation in the SC area has been conducted to ensure its reliability by comparing model outputs and ERA5 reanalysis data. For Case 2013, a pronounced 2-m temperature gradient is observed from the north of the SC region to the ocean in ERA5 (see Fig. S5a). Cold air is situated north of SC, while higher temperatures are evident in the GBA mega-city, reaching 13 °C—warmer than the surrounding rural areas. Simultaneously, prevailing northerly 10-m winds dominate the SC land area, leading to the intrusion of cold air into the region. Along the coast and over the ocean, these winds become northeasterly. The model's simulated surface temperature and wind patterns (see Fig. S5b) reasonably align with ERA5 reanalysis data. In addition, the model outputs also exhibit accurate low-level (925 hPa) temperature and wind simulations for the SC area compared to ERA5 reanalysis data (see Fig. S5c,d), demonstrating the model's reliability.

With regard to surface temperature and wind, the meteorological conditions in Case 2015 closely resemble those in Case 2013 (see Fig. S6). The model accurately reproduces the background 10-m winds in the SC area, demonstrating satisfactory performance in simulating the speed and direction of the northerly winds. Although the model slightly underestimates the 2-m temperature in the southern portion of SC compared to ERA5, the 2-m temperature performance in the GBA mega-city and the ocean remains reasonable. The model's output of 925 hPa temperature, wind speed, and wind direction in SC closely aligns with ERA5 data. In conclusion, the weather parameters of temperature, wind, and precipitation are well-simulated in the model for the two winter extreme events in the SC region, providing reasonable and comparable results compared to station observations. These results underscore the WRF model's ability to capture key meteorological features of the regional climate, providing a solid foundation for further analysis of urbanization impacts on extreme winter precipitation.

### Supplementary Information

Supplementary Figures.

## Data Availability

The Weather research and forecasting (WRF) model V4.3.3 coupled with single layer urban canopy model (SLUCM) was downloaded online (https://www2.mmm.ucar.edu/wrf/users/download/get_sources.html). ERA5 hourly data on pressure levels was from https://cds.climate.copernicus.eu/cdsapp#!/dataset/reanalysis-era5-pressure-levels?tab=form. ERA5 hourly data on single levels can be found in https://cds.climate.copernicus.eu/cdsapp#!/dataset/reanalysis-era5-single-levels?tab=form. TRMM-3B42 rainfall estimate products are from 10.5067/TRMM/TMPA/3H/7. Variables from WRF model output are available on Zenodo (10.5281/zenodo.8332470).
