# Supplementary material for: Analyzing urban influence on extreme winter precipitation through observations and numerical simulation of two South China case studies
Source: Sci Rep. 2024 Jan 24;14:2099. doi: 10.1038/s41598-024-52193-2 (PMC10808133; doi:10.1038/s41598-024-52193-2)
Supplement: Supplementary file 1 — Supplementary Figures. [file 41598_2024_52193_MOESM1_ESM.docx]

**Analyzing Urban Influence on Extreme Winter Precipitation through Observations and Numerical Simulation of Two South China Case Studies**

^1^Chenxi Hu, ^1,2^Chi-Yung Tam, ^3^Zong-liang Yang, ^4,5^Ziqian-Wang

^1^ Earth System Science Programme, the Chinese University of Hong Kong, Hong Kong, China

^2^Shenzhen Research Institute, the Chinese University of Hong Kong, Shenzhen, China

^3^Department of Geological Sciences, Jackson School of Geoscience, The University of Texas at Austin, Austin, TX, USA

^4^School of Atmospheric Sciences, Sun Yat-sen University, and Southern Marine Science and Engineering Guangdong Laboratory (Zhuhai), Zhuhai, China

^5^Guangdong Province Key Laboratory for Climate Change and Natural Disaster Studies, Sun Yat-sen University, Zhuhai, China

Submitted to Scientific Reports

November, 2023

Corresponding author address: Prof. Chi-Yung Francis Tam, Earth System Science Programme, the Chinese University of Hong Kong, Hong. Email: Francis.Tam@cuhk.edu.hk


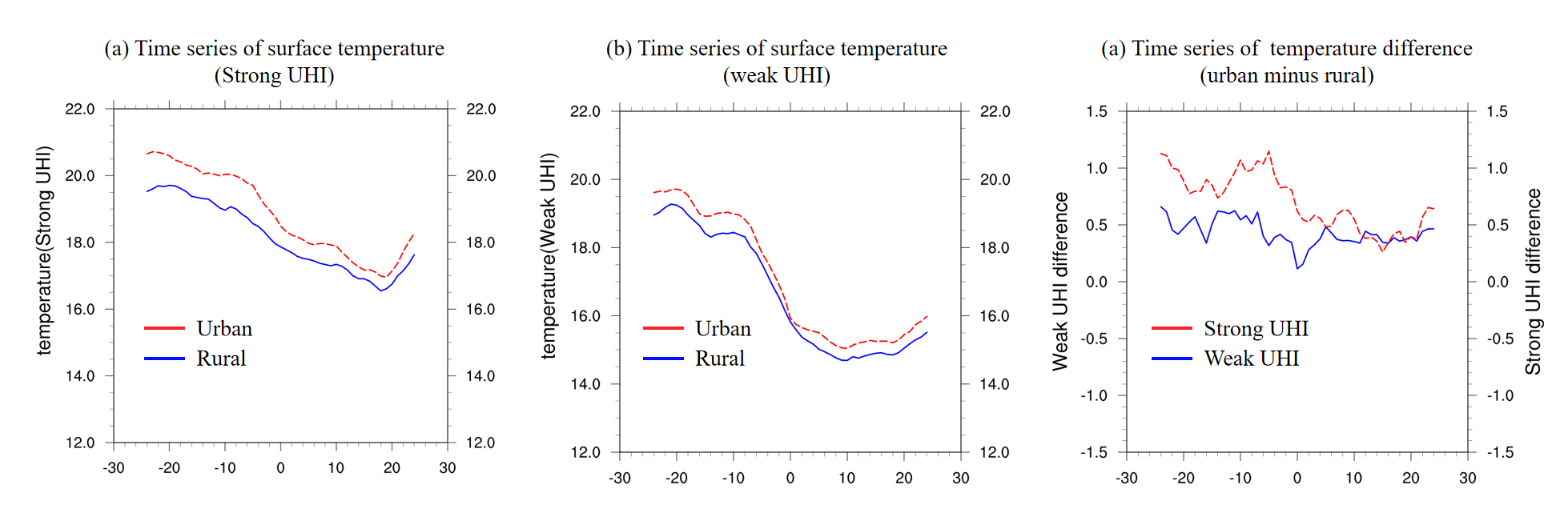


**Figure S1** (a) Time series of surface temperature (units: ^o^C) averaged over urban and rural stations during all strong UHI cases, with red and blue line representing temperature in urban and rural stations. Time 0 refers to the hour of peak rainfall averaged over the selected 14 stations. (b) Same as (a) except for surface temperature during weak UHI cases. (c) Time series of surface temperature difference (units: ^o^C) between urban and rural stations for strong and weak UHI cases, red and blue line mean results in strong and weak UHI cases.


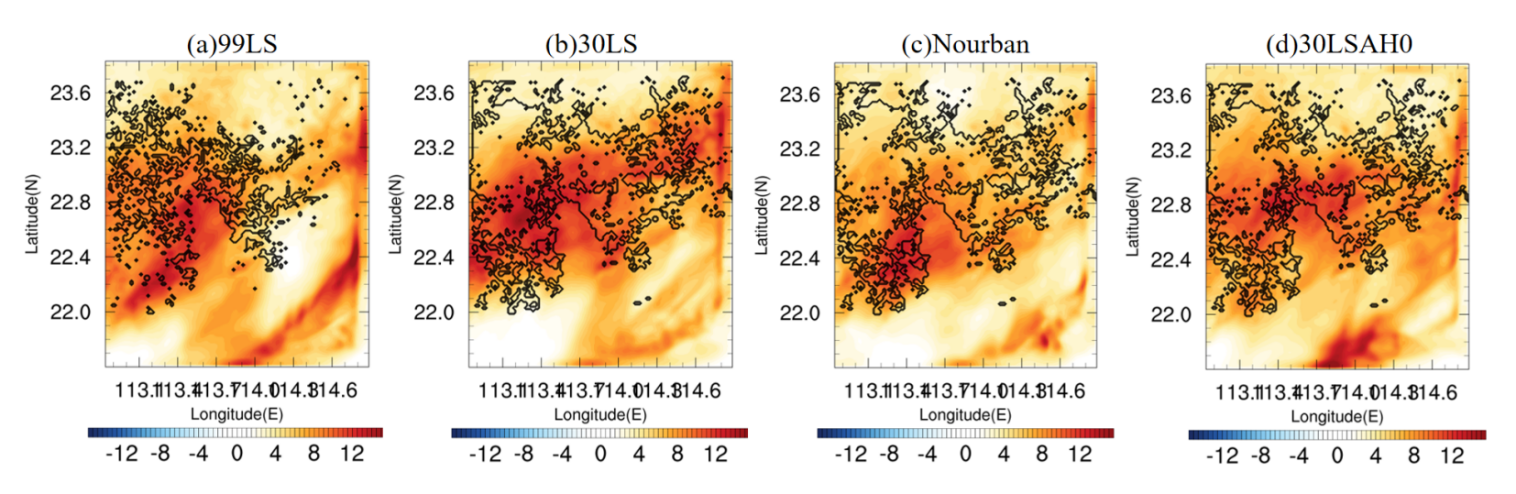


**Figure S2** Snapshots of hourly rainfall (units: mm/hr) distribution at the hour of urban peak rainfall for (a) 99LS, (b) 30LS, (c) Nourban, and (d) 30LSAH0. Black contours represent the boundary of the 1999 and 2030 GBA mega-city. The maps were generated by the NCAR Command Language (NCL) Version 6.6.2^80^, http://dx.doi.org/10.5065/D6WD3XH5.


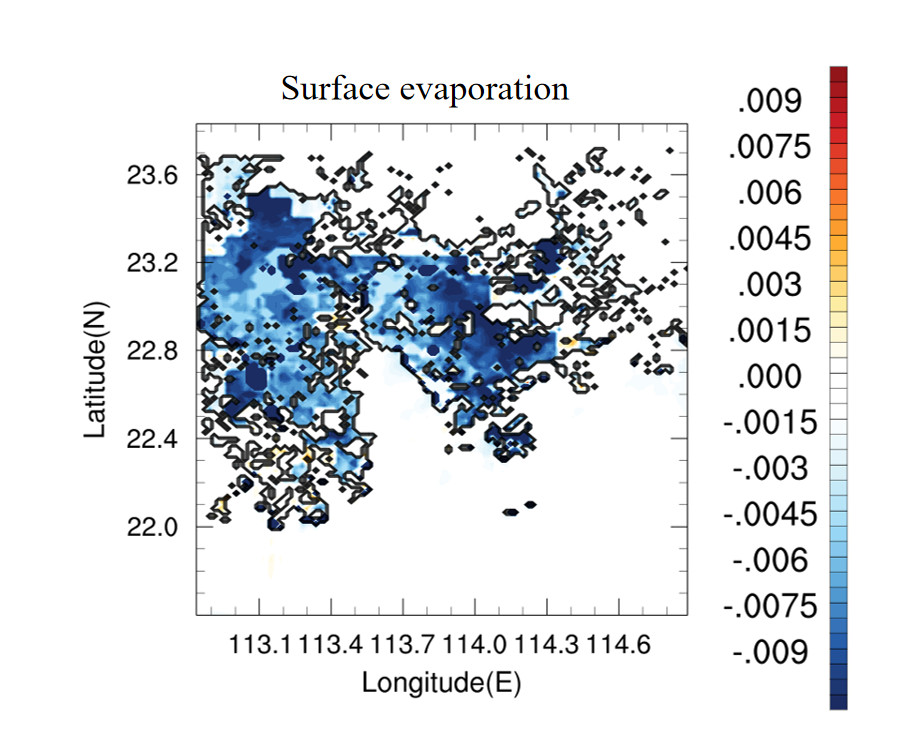


**Figure S3** Surface evaporation difference (units: g/m^2^/s) between 30LS and Nourban averaged over precipitation periods for Case 2013. Black contours represent the 2030 GBA mega-city. The maps were generated by the NCAR Command Language (NCL) Version 6.6.2^80^, http://dx.doi.org/10.5065/D6WD3XH5.


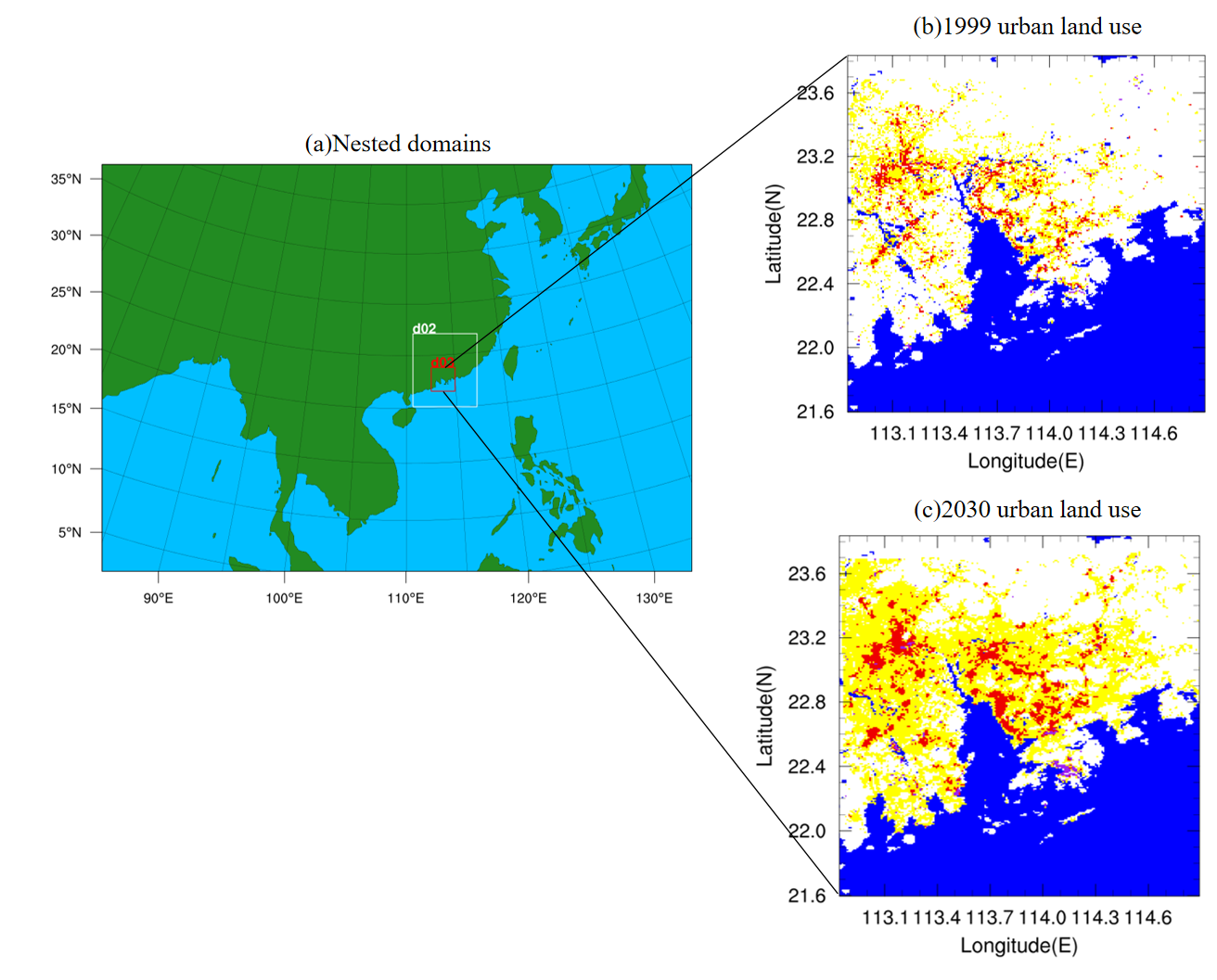


**Figure S4** (a) Nested domains for the WRF simulations. (b) Land use categories of 1999 in the innermost domain with “Low Intensity Residence”, “High Intensity Residence”, and “Commercial and Industrial” locations indicated by yellow, red, and purple shading. (c) Same as (b) but for projected 2030 urban land use. The maps of Nested domains were generated by the Weather research and forecasting (WRF) model Version 4.3.3^81^, <http://dx.doi.org/10.5065/1dfh-6p97>. And the maps of urban land use were generated by the NCAR Command Language (NCL) Version 6.6.2^80^, http://dx.doi.org/10.5065/D6WD3XH5.


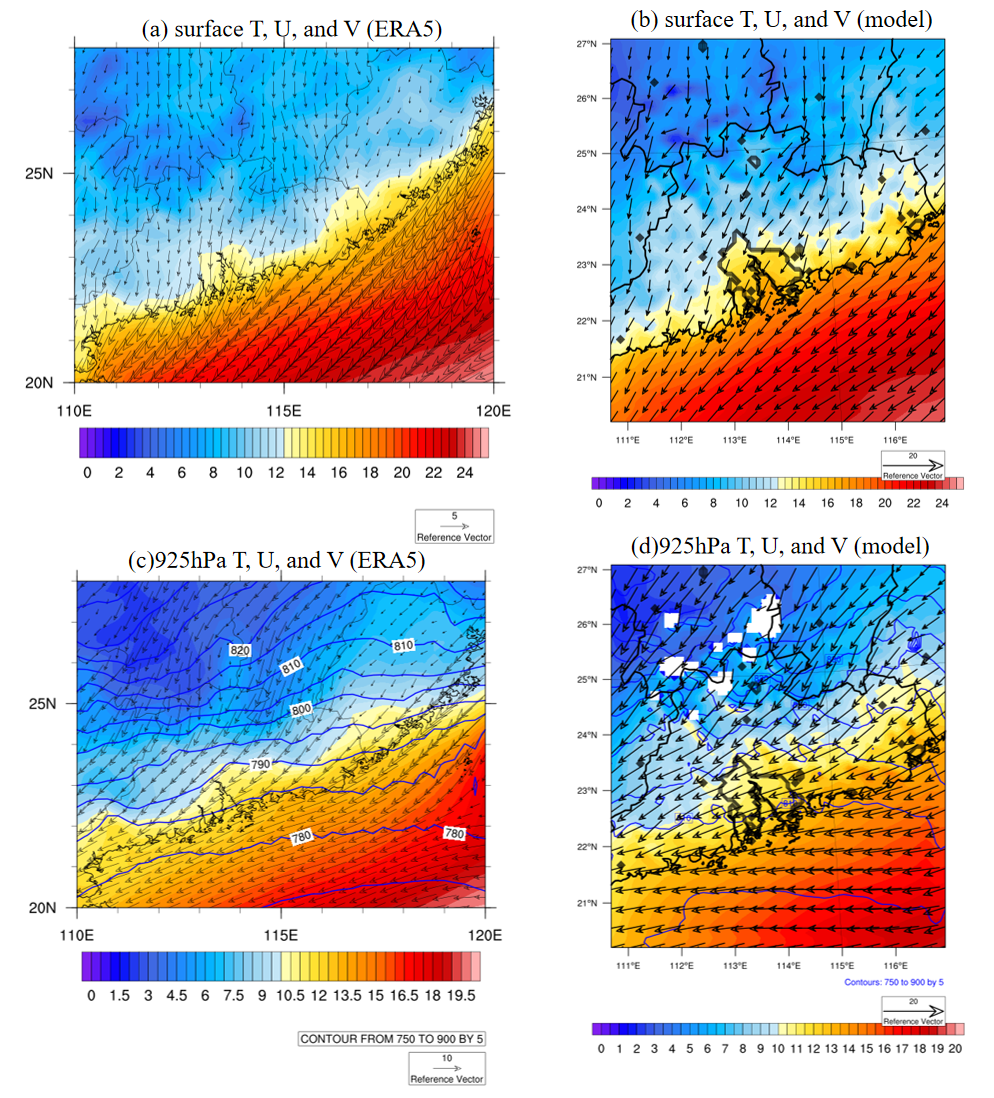


**Figure S5** (a) 2-m temperature (units: ^o^C), and 10-m U,V wind (units: m/s) averaged over Case 2013 from ERA5 reanalysis data, with shaded and vector representing temperature and wind. Black contours mean the coastline and boundary of province. (b) Same as (a) but for model outputs. (c) (d) Same as (a) (b) but for 925hPa level. The maps were generated by the NCAR Command Language (NCL) Version 6.6.2^80^, http://dx.doi.org/10.5065/D6WD3XH5.


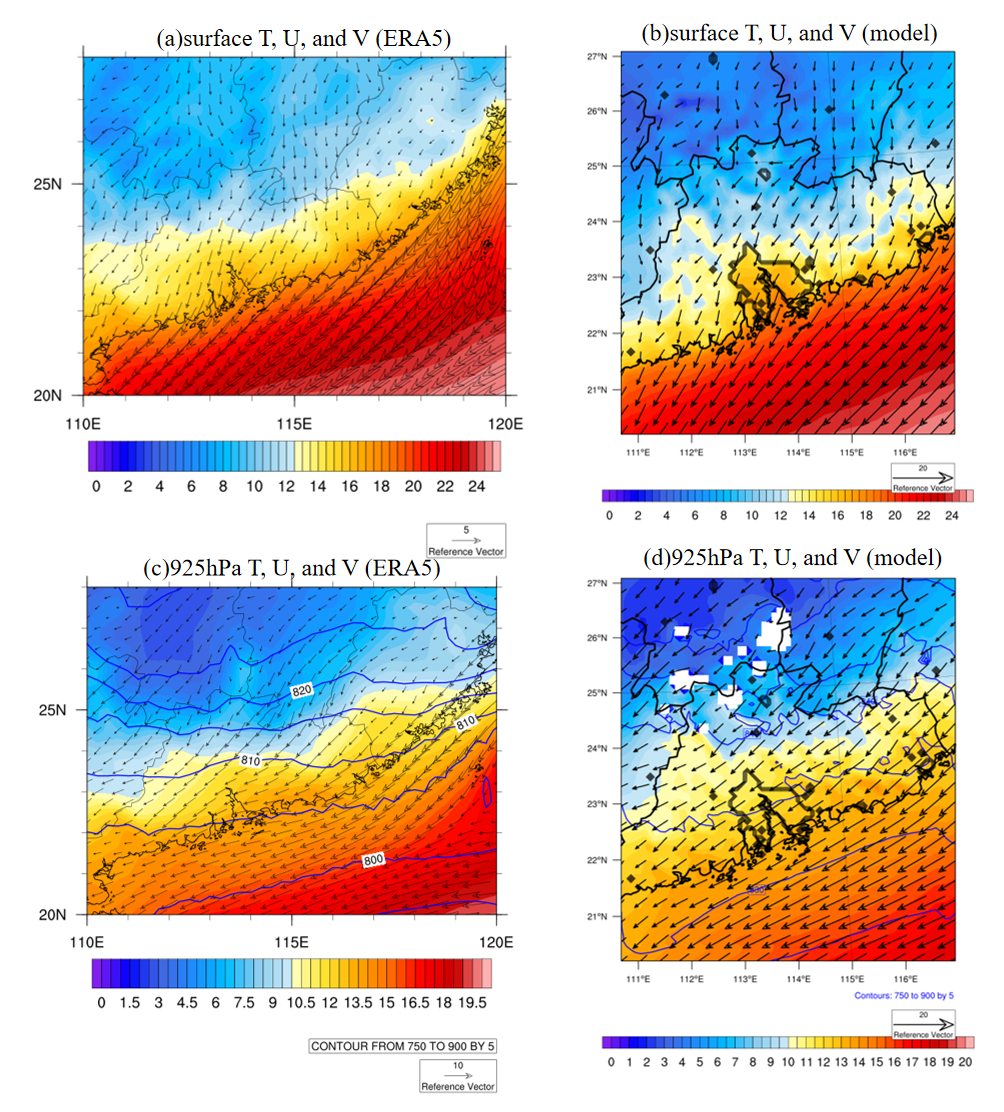


**Figure S6** Same as Figure S5 except for Case 2015. The maps were generated by the NCAR Command Language (NCL) Version 6.6.2^80^, http://dx.doi.org/10.5065/D6WD3XH5.
